# Quasiparticle Effects and Strong Excitonic Features in Exfoliable 1D Semiconducting Materials

**DOI:** 10.1021/acsnano.5c14061

**Published:** 2026-01-13

**Authors:** Simone Grillo, Chiara Cignarella, Friedhelm Bechstedt, Paola Gori, Maurizia Palummo, Davide Campi, Nicola Marzari, Olivia Pulci

**Affiliations:** † Dipartimento di Fisica, Università di Roma Tor Vergata and INFN, 00133 Rome, Italy; ‡ Max Planck Institute for the Structure and Dynamics of Matter (MPSD) and Center for Free-Electron Laser Science (CFEL), 22761 Hamburg, Germany; § Theory and Simulation of Materials (THEOS), and National Centre for Computational Design and Discovery of Novel Materials (MARVEL), École Polytechnique Fédérale de Lausanne, 1015 Lausanne, Switzerland; ∥ U Bremen Excellence Chair, Bremen Center for Computational Materials Science, and MAPEX Center for Materials and Processes, 9168 University of Bremen 28359 Bremen, Germany; ⊥ Institut für Festkörpertheorie und -optik, Friedrich-Schiller-Universität Jena, 07743 Jena, Germany; # Dipartimento di Ingegneria Industriale, Elettronica, e Meccanica, Università Roma Tre, 00154 Roma, Italy; ¶ Dipartimento di Scienza dei Materiali, Università degli studi di Milano Bicocca, 20126 Milan, Italy; ∇ Bicocca Quantum Technologies (BiQuTe) Centre, I-20126 Milan, Italy; ○ PSI Center for Scientific Computing, Theory and Data, 5232 Villigen, Switzerland

**Keywords:** semiconductor, chains, chalcogenides, one-dimensional, phonons, IR, excitons

## Abstract

We report a first-principles study of the electronic and optical properties of recently identified one-dimensional semiconducting materials exfoliable from van der Waals-bonded bulk crystals. Specifically, we investigate four chalcogenide-based atomic chains, the covalently bonded S_3_ and Te_3_ chains, and polar-bonded As_2_S_3_ and Bi_2_Te_3_ chains, using a fully first-principles approach that combines density functional theory (DFT), density functional perturbation theory (DFPT), and many-body perturbation theory within the *GW* approximation and Bethe–Salpeter equation (BSE). Our vibrational analysis shows that the isolated, freestanding wires remain dynamically stable, with the zone-center optical phonon modes leading to infrared activity. The main finding of this study is the presence of very strong exciton binding energies (1–3 eV), which make these exfoliable 1D materials suitable platforms for room-temperature excitonic applications. Interestingly, the exciton character remains Wannier–Mott-like, as indicated by average electron–hole separations greater than the lattice constant. Notably, the optical gaps of these materials span a wide range — from infrared (0.8 eV, Bi_2_Te_3_), through the visible spectrum (yellow: 2.17 eV, Te_3_; blue: 2.71 eV, As_2_S_3_), up to ultraviolet (4.07 eV, S_3_) — highlighting their versatility for broadband optoelectronic applications. Our results offer a detailed, many-body perspective on the optoelectronic behavior of these low-dimensional materials and underscore their potential for applications in nanoscale optoelectronic devices.

## Graphical abstract

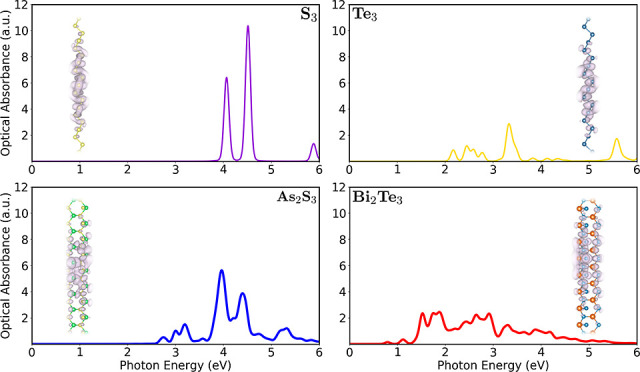

One-dimensional (1D) materials, such as nanotubes and nanowires, represent a rapidly evolving frontier in advanced materials research, owing to their exceptional electronic, optical, and mechanical properties. Their reduced dimensionality gives rise to distinct quantum phenomena that profoundly influence charge transport, light–matter interactions, and coupling with the surrounding environment. These effects position 1D materials as highly promising candidates for a wide range of technological applications, including nanoelectronics, spintronics, and sensing technologies.^1–3^ Among the most exciting directions is their use in optoelectronics, where the strong optical response of 1D systems — characterized by enhanced light absorption and tightly bound excitons — is relevant for the design of efficient photodetectors, lasers, and solar cells.

Several experimental techniques have recently been developed to obtain nanowires, including direct growth on a substrate,^4–6^ synthesis with directing agents,^7,8^ and encapsulation inside single-walled carbon nanotubes (CNTs).^9^ In recent years, a particularly promising strategy for accessing single nanowires has emerged, based on the exfoliation of strongly anisotropic three-dimensional (3D) crystals composed of covalently bonded inorganic chains that are held together by weak interchain interactions.^1,2^ In these materials, individual 1D wires are arranged in a crystal lattice via van der Waals (vdW) forces, allowing for their isolation through mechanical or chemical exfoliation techniques,^10–13^ analogously to the well-established methods developed for two-dimensional (2D) materials.

Such techniques have already been successfully employed to exfoliate quasi-1D vdW materials into individual wires or bundles with lateral dimensions down to ∼7 nm.^10,14,15^ For example, TaSe_3_ has been obtained by chemical–mechanical exfoliation, as in graphene and MX_2_ systems, and thin nanowires have also been realized by more scalable CVD growth. Beyond TaSe_3_, several other quasi-1D wires have been isolated by similar approaches,^12,16^ including TMTCs such as ZrTe_3_, HfSe_3_, and MoI_3_ ^11,17,18^ down to the single-chain limit.^19^ TiS_3_ has been exfoliated into single nanoribbons using AFM-assisted micromechanical exfoliation,^13^ while both mechanical and liquid-phase exfoliation have enabled quasi-1D ribbons in Ta_2_Pt_3_Se_8_ and Ta_2_Pd_3_Se_8_.^1,20,21^ Similar exfoliation strategies can likewise be applied to prepare single Te chains.^22^Quasi-1D vdW atomic chains have also been prepared by encapsulation in carbon or boron nitride nanotubes,^23–25^ as shown for TMTC nanowires,^26^ trigonal Te chains,^23,24^ and Sb_2_Se_3_,^27^ among others. CNT encapsulation both stabilizes the wires and protects them from oxidation, enabling the isolation of few-wire bundles or even single chains.

Unlike CNTs, exfoliated wires offer well-defined and reproducible electronic and optical properties, making them highly attractive for nanoelectronic applications. Their structural uniformity and reduced edge scattering have demonstrated significant promise as fundamental components in electronic devices, particularly field-effect transistors (FETs).^10,11,14,20^ Moreover, their anisotropic bonding and the absence of dangling bonds perpendicular to the wire axis — exemplified in materials such as BiSI, BiSeI, Sb_2_Se_3_, and Bi_2_Se_3_ — can be used to enhance charge carrier lifetimes and minimize nonradiative recombination, thus boosting the performance of high-efficiency photoconversion devices.^28,29^ CNTs and semiconductor nanowires typically exhibit diameters in the range of a few nanometers, where electronic wave functions retain a quasi-3D character and the screening remains partially effective.^30^ Instead, in the (quasi-)­1D limit, due to the significant confinement of the inhomogeneous electron gas and the strong reduction of its screening, Coulomb interactions are enhanced and, consequently, many-body exchange-correlation effects become more dramatic than in nearly free-electron-like 3D materials. This holds especially for electronic excitations.

Computational high-throughput (HT) screening offers a powerful tool to discover interesting one-dimensional wires. Such studies have been effective in identifying 3D materials suitable for exfoliation in atomically thin 2D layers,^31–38^ and recently similar approaches have been applied to identify one-dimensional systems.^3,39–42^ Recently, Campi and coauthors created a large data set of 1D systems that can be exfoliated from weakly bonded quasi-1D crystals.^43,44^ This 1D database is built by screening three different 3D databases (COD,^45^ ICSD,^46–48^ and Pauling File^49^) containing all structures experimentally reported in their 3D form.

In this study, we target exfoliable 1D wires promising for future technological applications, with a particular focus on optoelectronic applications. To date, investigations on feasibly exfoliable 1D materials, using state-of-the-art MBPT methods, have been fairly sparse.^50–53^ Here, we aim to predict electronic and optical properties and understand them in terms of dimensionality, enhanced electron–electron interaction, and quantum confinement. We select promising materials from the above database of exfoliable 1D systems,^43,44^ according to the following criteria: we screen wires (i) mechanically stable from the calculated phonon dispersions, (ii) with a maximum of two atomic species per unit cell, and (iii) with semiconducting behavior. We further screen for (iv) a DFT direct electronic band gap below 3 eV. We finally select four chalcogenide-based materials, namely S_3_, Te_3_, As_2_S_3_, Bi_2_Te_3_ [Fn fn1].

To tackle these materials and the calculation of their properties, we employ density-functional theory (DFT) and many-body perturbation theory (MBPT), including *G*_0_ *W*_0_, eigenvalue self-consistent *GW* (*evGW*) and the Bethe–Salpeter equation (BSE).

## Results and Discussion

### Structural and Vibrational Properties

We begin our analysis by characterizing the structural properties of the 1D materials investigated in this work. These have been obtained through HT exfoliation of weakly bonded 3D parent compounds,^43,44^ sourced from databases containing experimentally reported structures only. In particular, we investigate chain structures of S_3_, Te_3_, As_2_S_3_, and Bi_2_Te_3_ (see Figure 1), which can be exfoliated from S_9_ ^54,55^ (S_3_), α-Te^56–58^ (Te_3_), As_8_S_12_ ^59,60^ (As_2_S_3_) and Bi_4_Te_6_ ^61,62^ (Bi_2_Te_3_). In light of recent mechanical exfoliation of Bi_2_Te_3_ thin layers^63,64^ and the robust ambient-environment stability observed in related 2D As_2_S_3_ nanosheets^65,66^ and Te nanowires,^22^ we emphasize that the 1D compounds studied here share the same van der Waals structural motif and weak interchain cohesion, making their experimental exfoliation and stability highly plausible.

**Figure 1:**
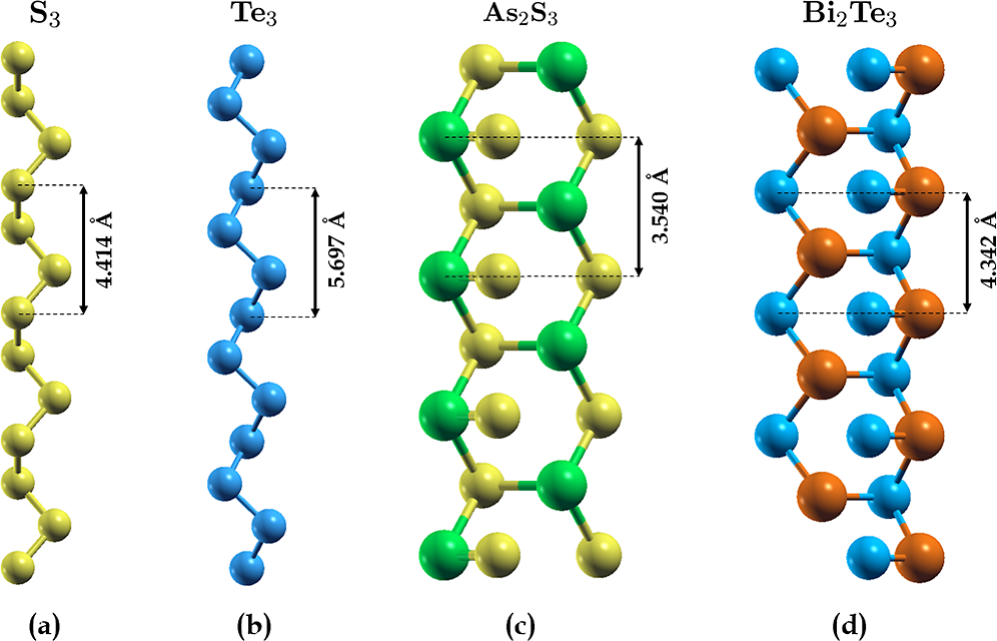
Optimized geometric structures of (**a**) S_3_, (**b**) Te_3_, (**c**) As_2_S_3_ and (**d**) Bi_2_Te_3_. The optimized lattice parameters of the unit cells are also displayed.

We perform on each material an additional full-structure relaxation to obtain the optimized lattice parameters and atomic positions using the chosen XC functional. The resulting values are reported in Table 1. The structures of S_3_ (Figure 1a) and Te_3_ (Figure 1b) both consist of a chiral helical chain composed of three atoms in the unit cell, arranged so that the projection of the chain onto *a* plane perpendicular to the chain axis forms an equilateral triangle. Such a structure is typical of chalcogen elements, even for higher-dimensional arrangements. The unit cell of As_2_S_3_ (Figure 1c) consists of two As atoms and three S atoms, which are alternately bonded to form a network of nonplanar hexagons. Additionally, there is an extra S atom attached to each hexagon (through an As atom), protruding toward its center. The structure of Bi_2_Te_3_ (Figure 1d) is specular to that of As_2_S_3_, with Bi and Te replacing As and S, respectively. The described geometries give rise to minima on the total energy surface, thereby indicating the static energetic stability.

**Table 1:** Optimized Lattice Parameter (*a*) of the Systems Studied, Obtained by Full-Structure Relaxation Using a GGA-PBE XC Functional, Together With the Calculated Lowest Electronic Gap (*E*_g_^DFT^) at the DFT Level^a^

	**S**_3_	**Te**_3_	**As**_2_ **S**_3_	**Bi**_2_ **Te**_3_
*a* (Å)	4.414	5.697	3.540	4.342
*E*_g_^DFT^(eV)	2.66 [2.76]	1.43 [1.47]	1.16 [1.26]	0.42 [0.43]

a In the case of indirect band gap, the corresponding direct band gap is also reported in square brackets. SOC was included.

The equilibrium geometries obtained (see Table 1) are used as starting structures for the investigation of the vibrational properties. We compute the phonon dispersion curves along the 1D Brillouin zone (BZ), between the center Γ and the boundary point *Z*, using finer **k**- and **q**-points grids, as well as larger vacuum space and stricter energy and forces thresholds with respect to the calculations performed in the source HT study (which inherently requires a compromise between accuracy and computational cost). The results are shown in Figure 2: S_3_ Figure 2, Te_3_ (Figure 2b) and Bi_2_Te_3_ (Figure 2d) exhibit no imaginary phonon frequencies along the entire BZ. Therefore, these chain-like structures are also dynamically stable in their freestanding configuration as exfoliated from their 3D counterparts. We only note a small instability in As_2_S_3_ Figure 2c). The lowest transverse acoustic (TA) phonon branch exhibits imaginary (negative in the plot) frequencies around 2/3 Γ*Z*. This fact may be interpreted as a tendency for a reconstruction toward a tripling of the unit cell size. However, we stress that the calculations are performed in the harmonic approximation and at T = 0 K, therefore without accounting for the effect of anharmonicity and/or temperature, which could contribute to the stability of 1D systems.^67–70^

**Figure 2:**
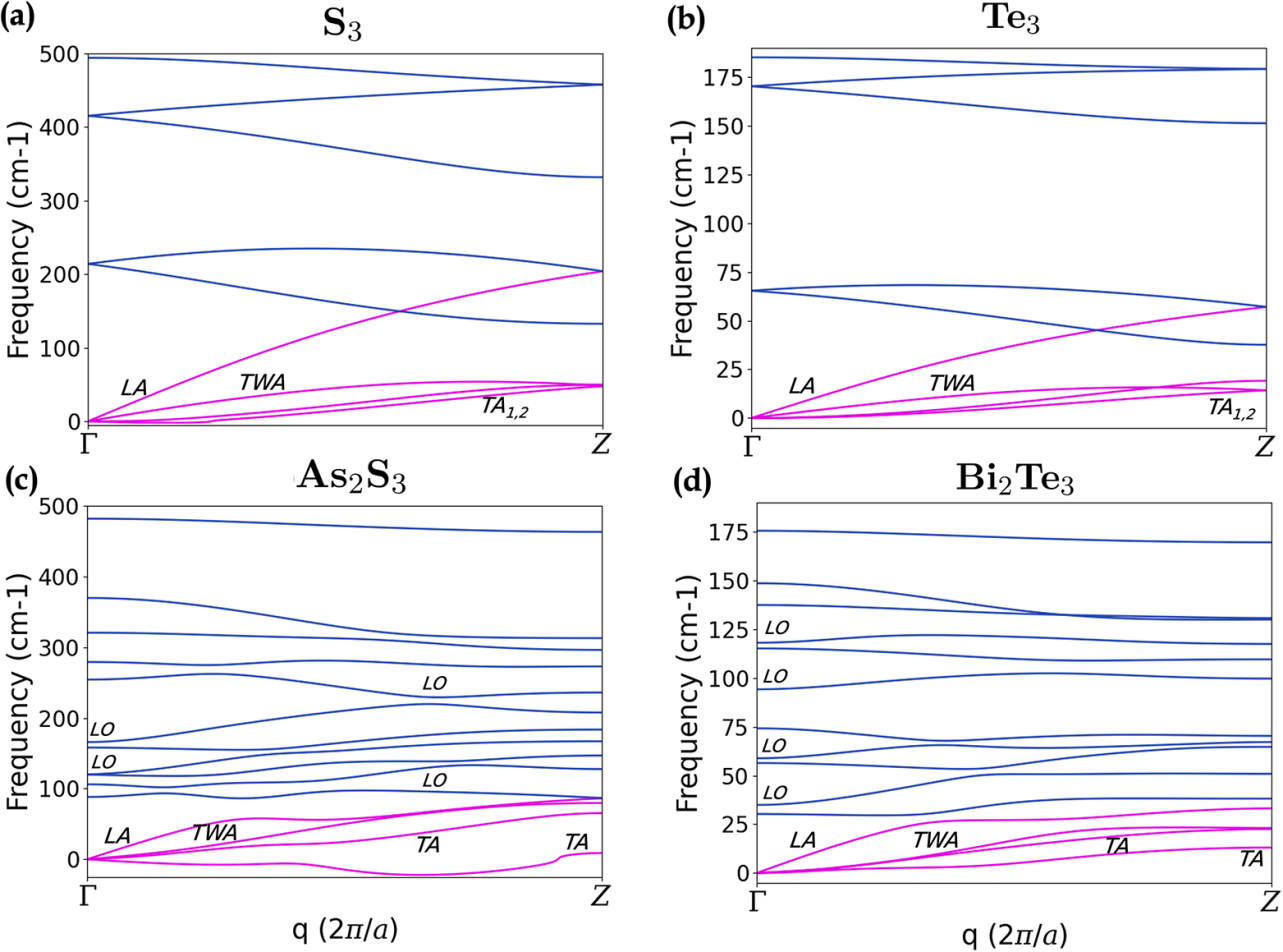
[Top] Phonon band structures of (**a**) S_3_, (**b**) Te_3_, (**c**) As_2_S_3_ and (**d**) Bi_2_Te_3_, calculated at harmonic level within DFPT. [Bottom] Top-view of (from left to right) S_3_, Te_3_, As_2_S_3_, Bi_2_Te_3_ with arrows indicating the displacements produced by TWA at Γ.

The phonon dispersions highlight four acoustic branches going to zero at Γ, outlined in magenta in Figure 2. In addition to the three translational modes, 1D wires are also invariant under rotation around their axis;^71,72^ this means that when completing a full rotation around it, the force acting on the wire is zero, and hence the corresponding phonon frequency is also zero. We denote this mode as twisting acoustic mode (TWA); it is illustrated in Figure 2 for the four materials, where this vibration appears as a rotation or twisting of the nanowire. The two acoustic branches quadratic in **q**, particularly visible in Figure 2a,b, are flexural modes representing transverse vibrations of atoms along the two directions perpendicular to the wire (TA) — similar to the ZA mode in 2D monolayers — which generate a bending of the wire. The highest acoustic mode is instead linear in **q**, and corresponds to longitudinal in-line vibrations (LA). Among the optical modes, the highest phonon branches of S_3_ and Te_3_ are associated with breathing modes, where atoms vibrate outward and inward from the center of the chains. Similar vibrational patterns are observed in As_2_S_3_ and Bi_2_Te_3_ in the 2 s-highest optical branches, while the highest isolated branch visible in Figure 2c,d represents breathing vibrations involving only the extra S (Te) attached to the hexagonal body.

In Figure 3 the IR absorption spectra resulting for the four wires are displayed. As_2_S_3_ (Figure 3c) and Bi_2_Te_3_ (Figure 3d) exhibit two main IR-active peaks, that couple to the atomic vibrations illustrated in the insets of the same figure. All their observable IR-active peaks correspond to LO (longitudinal optical) modes, with the oscillation strength aligned with the direction of propagation (ẑ) ${\mathrm{Z̅}}^{*}\cdot q_{z}\neq 0$ ,^73,74^ and labeled LO in Figure 2. Such LO modes are expected to shift from the closest TO (transverse optical) modes visible in the phonon dispersions. This effect is due to the long-range coupling between the macroscopic electric field generated by the longitudinal vibrations and the vibration itself, which in 3D results in the so-called LO-TO splitting between the LO and TO degenerate modes (without the field) at Γ.^75^ In 1D, the LO and the two TO modes are symmetrically nonequivalent and therefore not necessarily degenerate, and the long-range polar interaction produces not a splitting, but a blue-shift in frequency of the LO mode.^76^

**Figure 3:**
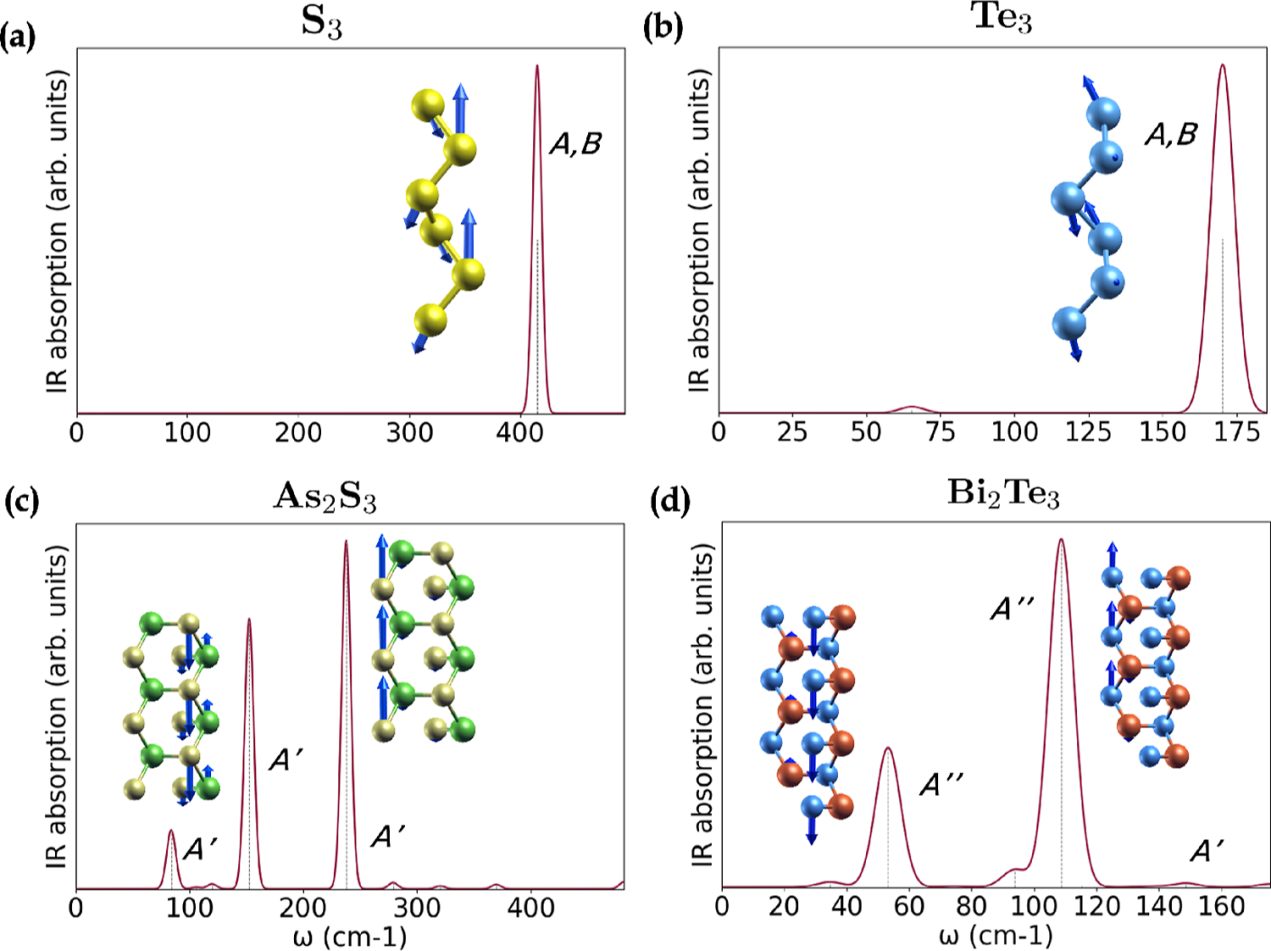
IR absorption spectra of (**a**) S_3_, (**b**) Te_3_, (**c**) As_2_S_3_ and (**d**) Bi_2_Te_3_, calculated at harmonic level of theory in DFPT. Insets show the corresponding vibrations of the main IR-active modes. The colors of the atoms are yellow (S), blue (Te), green (As) and red (Bi).

S_3_ (Figure 3a) and Te_3_ (Figure 3b) chains exhibit IR spectra similar to each other, consisting of two degenerate IR-active modes with identical vibrational patterns. In the inset, we show the *A* and *B* modes, respectively for S_3_ and Te_3_ (the vibrational patterns associated with each mode are then equivalent in both materials). Due to their similar geometry, only the frequencies of the phonon modes — and hence the peak positions — are (drastically) influenced by the nature of the element. For S_3_ the peak is shifted toward higher frequencies compared to Te_3_ by a factor of 2.4, which is only slightly larger than the square root of the mass ratio $\sqrt{M_{Te}/M_{S}}=2$ . Interestingly, the IR response of these modes arises from vibrations along *x̂* and *ŷ* directions and thus not classified as LOs: no shift or split around Γ is expected if the long-range polar effects are included in the calculation.^76,77^ Moreover, these two materials are homopolar, i.e., composed of the same atomic species, hence the polarization responsible for the IR peak does not derive from a difference in electronegativity of the atomic species, but rather from their structure asymmetry. This yields ta lower charge disproportion and a weaker IR response, as shown in Figure 4. Here, we compare the relative peaks of the four nanowires, normalized by their quantum volume: S_3_ and Te_3_ response is almost negligible as compared to that of As_2_S_3_ and Bi_2_Te_3_, the latter giving rise to the most intense peaks. Their dipole coupling makes their intensity greater than the homopolar cases by two to three orders of magnitude.

**Figure 4:**
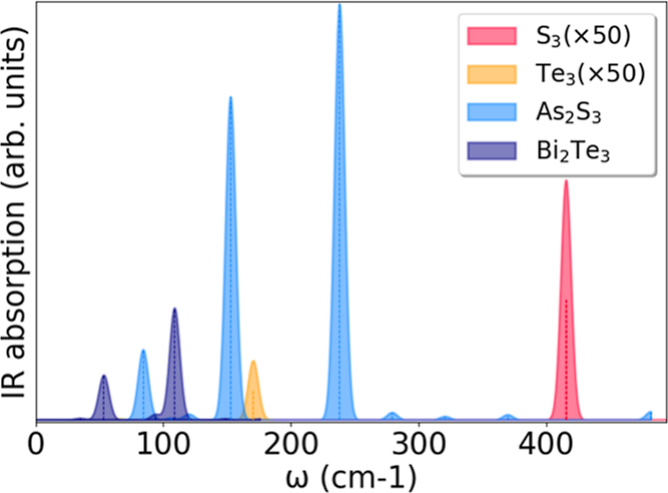
IR absorption peaks of the four nanowires. The IR response of S_3_ and Te_3_ is multiplied by a factor of 50 in the plot, as extremely weak (and otherwise indistinguishable) compared to the most prominent peaks of As_2_S_3_ and Bi_2_Te_3_.

The IR peaks in As_2_S_3_ and Bi_2_Te_3_ spectra in Figure 3 appear[Fn fn2] at zone-center frequencies, much below the uppermost S_3_- or Te_3_-dominated frequencies around 500 and 175 cm^–1^, respectively (see also Figure 2).

### Electronic and Optical Properties

In this section, we start by analyzing the electronic band structures of the four different materials under study, first obtained at the DFT level and then refined using QP corrections within the *GW* approximation. We then study their optical spectra at the highest many-body level, thus including the electron–hole (*e*–*h*) interaction by solving the BSE. Given their dimensionality, these materials exhibit exceptionally flat bands (especially S_3_ and Te_3_), which may harbor a range of exotic properties yet to be investigated, while also complicating a clear identification of the electronic band gap. In particular, these flat bands, together with the band degeneracies and the variation of the position of the band extrema in the BZ, make an effective-mass approximation near the VBM and the CBM difficult. We will return to this point in the following (Section *Understanding 1D excitons*). We highlight that, while our study addresses freestanding wires with pristine compositions, deviations from ideal stoichiometry — such as those discussed in ref 78 — as well as the influence of the substrate can alter the observed electronic and optical response, as further discussed in this and the following section.

The QP band structures, calculated within the evGW approach, are reported in the right panels of Figures 5 and 7, whereas the DFT band structures are shown in Figures 4 and 5 in the Supporting Information, without and with the inclusion of SOC, respectively. We refer the reader to the Supporting Information for a thorough analysis of these results. Here, we simply point out that all four wires are indirect semiconductors, with band gaps spanning from 0.4 to 2.7 eV. The corresponding values are listed in Table 1.

**Figure 5:**
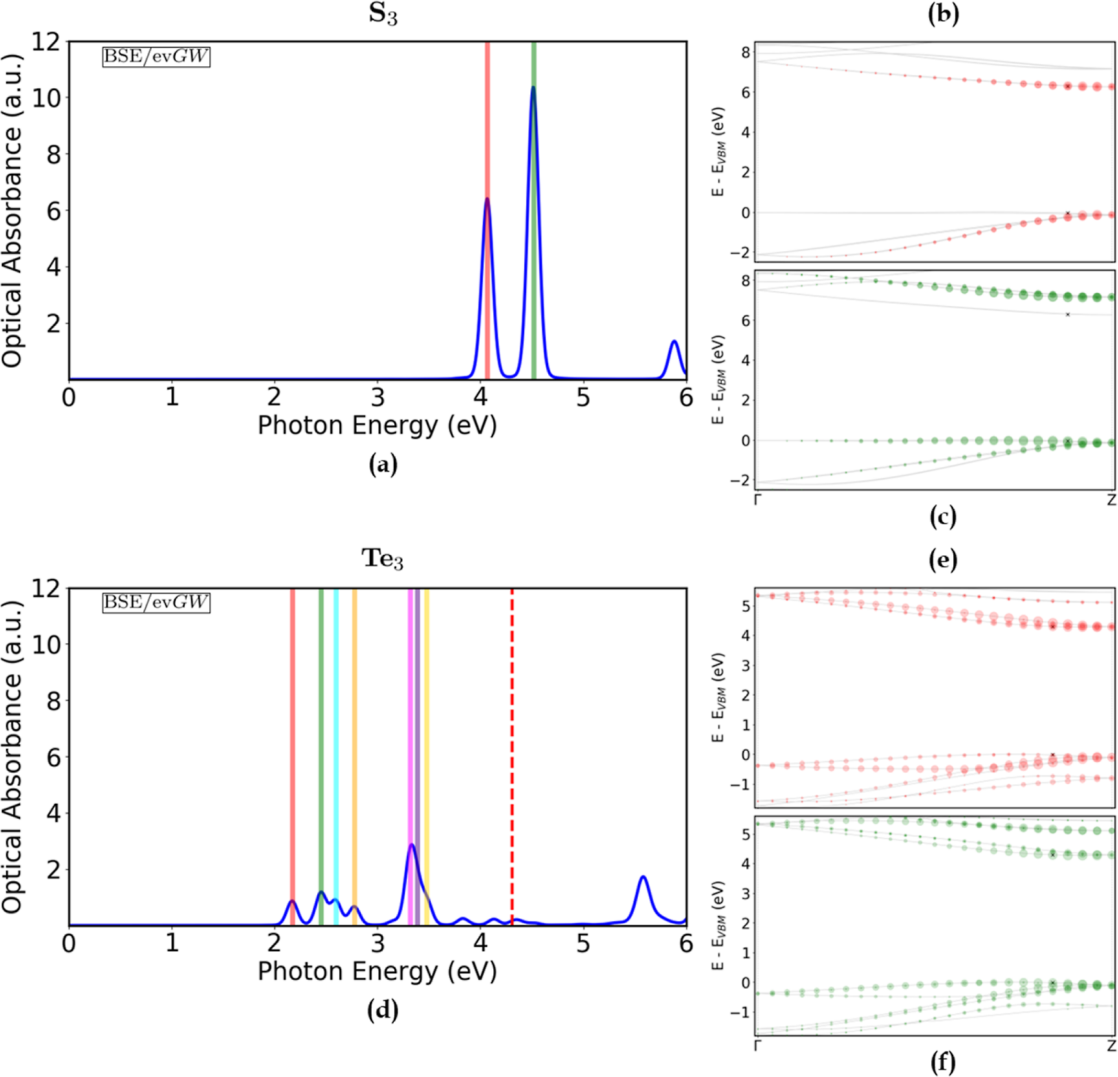
[Left] Absorption spectra (solid blue) of (**a**) S_3_ and (**d**) Te_3_, expressed in terms of the optical absorbance *A*(ω), calculated at the ev*GW*/BSE level. The corresponding ev*GW* – corrected direct electronic band gaps (dashed red) are shown as a reference (6.34 eV for S_3_). A broadening of 50 meV was used. [Right] Electronic band structures (solid gray), calculated at the ev*GW* level, of S_3_ (**b**,**c**) and Te_3_ (**e**,**f**). The colored dots (red and green) represent the single-particle transitions contributing to the first two bright excitons, and their size is proportional to the intensity of the transition - renormalized to the highest value. The corresponding excitonic peaks are highlighted in the relative absorption spectra (solid red and green), together with other meaningful higher excitations below the electronic gap (see Figure S12 in the Supporting Information). Energy zero is set as the top of the valence bands. SOC and semicore corrections were included.

Concerning the QP corrections, we find that GW results are significantly affected by the strong dependence of the empty states on the cell vacuum, which hinders the convergence of most key quantities in MBPT. For further details, we refer to the Supporting Information.

The extremely low screening in freestanding 1D systems necessitates the use of a methodology similar to that used for systems with a 3D confinement. For instance, to address this, single-shot *G*_0_ *W*_0_ calculations are followed by eigenvalue self-consistent calculations on both *G* and *W* (referred to as ev*GW*). The calculated values are presented in Table 2 and in Table 1 of the Supporting Information. The lowest electronic gaps are corrected to 5.46 (*G*_0_ *W*_0_) and then to 6.25 eV (ev*GW*) for S_3_; from 3.47 (*G*_0_ *W*_0_) to 4.27 eV (ev*GW*) for Te_3_; for As_2_S_3_, from 2.26 (*G*_0_ *W*_0_) to 3.00 eV (ev*GW*); finally, for Bi_2_Te_3_, from 1.54 (*G*_0_ *W*_0_) to 1.65 eV (ev*GW*). Remarkably, in the latter case the electronic band gap undergoes an indirect-to-direct transition, as the QP correction at Γ ends up being higher than those at neighboring **k**-points. Upon convergence, the QP gaps are increased by the ev*GW* corrections by 3.59 (S_3_), 2.84 (Te_3_), 1.74 (As_2_S_3_), and 1.23 (Bi_2_Te_3_) eV according to the decreasing fundamental gap and, therefore, the increasing electronic polarizability.

**Table 2:** Calculated Lowest Direct Electronic Band Gaps (*E*_g_^evGW^), at the ev*GW* Level, Together with the BSE/ev*GW* Optical Gaps (*E*_opt_^BSE/evGW^) and the Corresponding Binding Energies (*E*_b_^BSE/evGW^) and Radii (*r*_b_^BSE/evGW^) of the Lowest Bright Excitons^a^

	*E*_g_^evGW^(eV)	*E*_opt_^BSE/evGW^(eV)	*E*_b_^BSE/evGW^(eV)	rbBSE/evGW(A°)	*E*_b_^M^(eV)	rbM(A°)
**S**_3_	6.25 [6.34]	4.07	2.27 (1.93)	12.4 (12.5)	1.80/2.19	6.4/6.0
**Te**_3_	4.27 [4.31]	2.17	2.14 (1.65)	14.7 (14.8)	1.62/1.94	7.7/7.2
**As**_2_ **S**_3_	3.00 [3.04]	2.71	0.33 (0.89)	14.4 (12.00)	1.24/1.47	11.2/10.5
**Bi**_2_ **Te**_3_	1.65	0.80	0.85 (0.77)	13.2 (13.2)	0.78/0.94	16.7/15.5

a In the case of an indirect QP band gap, the corresponding direct band gap is also reported in square brackets. In round brackets, the corresponding BSE/*G*_0_ *W*_0_ findings can be found for comparison. The last two columns show the exciton binding energies (*E*_b_^M^) and radii (*r*_b_^M^) obtained by the analytical model described later (Section *Understanding 1D excitons*), using the softcore/modified softcore 1D hydrogen potential. The SOC and semi-core corrections were included. An extended table is reported in the Supporting Information.

The absorption spectra of the four 1D systems, calculated at the BSE level using ev*GW*-corrected QP states, are presented in Figures 5 and 7. For comparison, the same spectra derived from *G*_0_ *W*_0_-corrected states are shown in Figures 6 and 7 of the Supporting Information. For the sake of brevity, here we focus our discussion on the ev*GW*/BSE spectra. The resulting optical band gaps are reported in Table 2, together with the binding energies and radii of the lowest bright excitons. The optical absorption is expressed here in terms of the frequency-dependent optical absorbance (or absorption coefficient) *A*(ω) = (ω/*c*) Im­[α_1D_(ω)], where we have defined the 1D macroscopic electronic polarizability of the wire as α_1D_(ω) = (ε­(ω) −1) *S*/(4π), with ε­(ω) the dielectric function and S the surface area of the supercell transverse to the axis of the wire, thus making the intensity of the spectra independent of the supercell vacuum. The optical absorbance predicted should be experimentally measurable both for freestanding atomic wires and for van der Waals-bound stacks of wires. In the case of substrates or encapsulated configurations, the predicted spectral behavior should remain observable as long as the characteristic absorption edges of the surrounding materials lie above those of the studied atomic wires. In such cases, the spectral intensity may vary due to environmental screening and dielectric effects.

**Figure 6:**
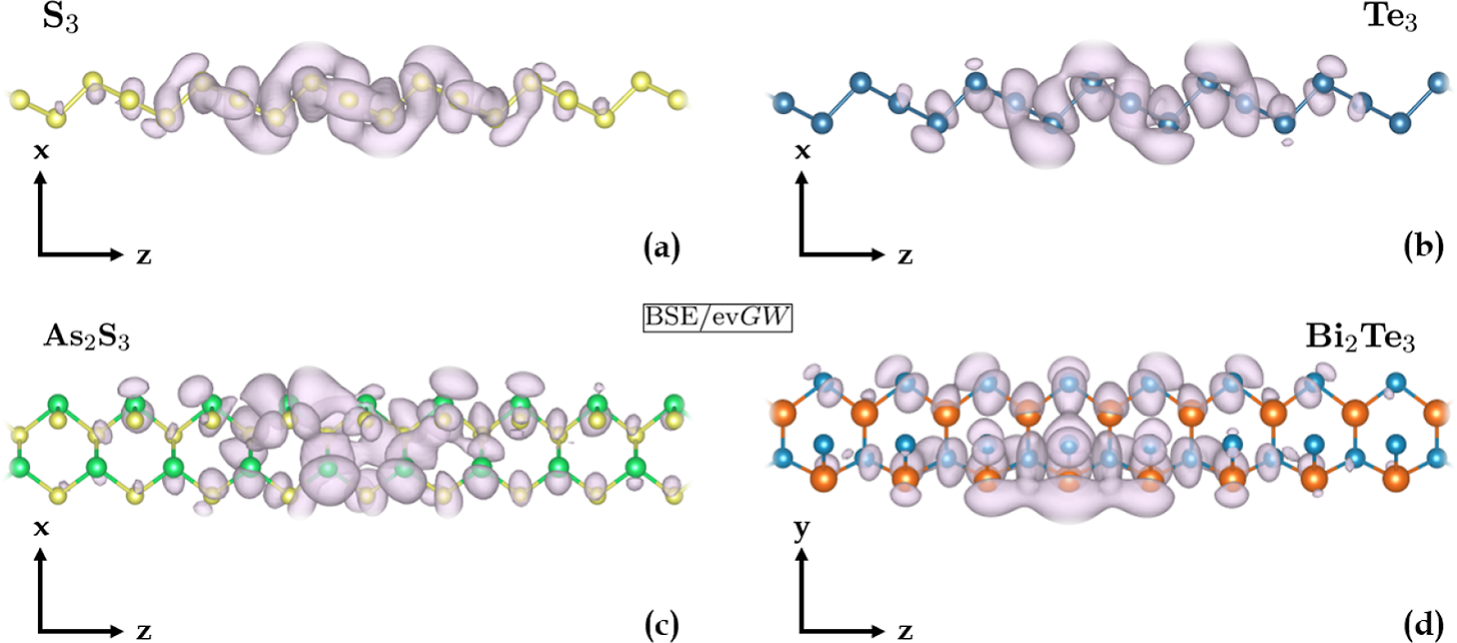
Plots in direct space of the excitonic wave functions of the first bright exciton of (**a**) S_3_, (**b**) Te_3_, (**c**) As_2_S_3_ and (**d**) Bi_2_Te_3_, calculated at the ev*GW*/BSE level. The excitonic wave function plot describes the probabilty of finding the electron in the exciton once the position of the hole is fixed. Its extent along the *z*-axis is quantitatively related to the exciton radii of Table 2. The position of the hole was chosen based on the localization of the valence electrons contributing to the exciton. The number of cell repetitions in the periodic direction was increased until the wave function decayed to zero.

The analysis of the results obtained from the BSE calculations is divided into two parts, based on the common features of the four materials considered. We first investigate the two elemental wires S_3_ and Te_3_. As shown in Figure 5, the spectra of these two systems, below and around the direct QP gap, consist of relatively intense optical excitations that are well-separated in energy, resembling what one might expect from strongly confined systems. Below the gap, they represent dipole-allowed excitons, whose intensities and excitation energies do not follow a simple law in the main quantum number. With an optical gap of 4.07 (S_3_) and 2.17 (Te_3_) eV, the lowest bright excitations are located in the near-UV and in the visible range, respectively. The lowest exciton bound states are much below the QP gaps in Table 2. Consequently, they exhibit a significant binding energy above 2 eV.

In the case of S_3_ (Figure 5a), two prominent excitations appear below the direct electronic band gap (6.34 eV), followed by a smaller peak. They are bound excitonic states with varying binding energy. The first exciton, which marks the onset of optical absorption, occurs at 4.07 eV, with a binding energy of 2.27 eV. As shown in Figure 5b, it arises from transitions involving the third highest degenerate occupied band and the first (degenerate) conduction band, close to the *Z* point. The corresponding excitonic wave function, whose radius (i.e., its lateral extent), is estimated to be 12.4 Å, is depicted in Figure 6a. The spatial extent of the excitonic wave function indicates that, despite the large binding energy, the exciton preserves a Wannier–Mott-like character. The second and most intense peak (4.51 eV) instead stems from transitions involving mainly the doubly degenerate valence band and the doubly degenerate second conduction band, again close to the *Z* point, as illustrated in Figure 5c.

In the case of Te_3_ (Figure 5d), the optical absorption spectrum consists of several bright excitations below the direct electronic band gap (4.31 eV). Tellurium has been extensively investigated in recent years. Particularly, 2D tellurium — known as tellurene —, found in different allotropic forms,^79^ has emerged as a promising semiconductor with a thickness-tunable band gap,^80^ relatively high carrier mobility^81,82^ and remarkable electronic and optical characteristics, with optical band gaps ranging from 0.84 to 1.46 eV and a light absorption as high as 50%.^83^ 3D tellurium is instead a narrow-gap semiconductor, with an estimated exciton binding energy of less than 10 meV.^84^ In its 1D counterpart, the first bright exciton appears at 2.17 eV, thus with a remarkable binding energy of 2.14 eV. It is noteworthy to mention that previous calculations done at the *G*_0_ *W*_0_/BSE level predict for Te_3_ a similar exciton binding energy of 2.07 eV^84^ and 2.35 eV,^51^ with a *G*_0_ *W*_0_ direct gap of, respectively, 4.23 and 4.44–4.59 eV, surprisingly close to our ev*GW* estimated direct gap of 4.31 eV. The apparent agreement in the gap is related to the missed inclusion of SOC in both the aforementioned calculations, leading to an overestimation of the QP corrections. The exciton binding energy, however, is practically not affected by SOC. The transitions contributing to this peak involve the six uppermost valence bands, and the three lowest conduction bands, with major contributions coming from the second highest valence band and the second lowest conduction band, spanning the entire BZ (Figure 5e). The corresponding excitonic wave function, with a Bohr radius of 14.7 Å, is depicted in Figure 6b, closely resembling that of S_3_. The second bright exciton, occurring at 2.45 eV, involves the same bands present in the previous one, with a slightly different distribution of the single-particle contributions (Figure 5f). Other meaningful higher bound states, highlighted in Figure 5d by different colors, are found below the electronic gap. The contributions to these peaks are illustrated in more detail in Figure S12 in the Supporting Information and will not be discussed here.

In contrast to the elemental cases, the absorption spectra of As_2_S_3_ and Bi_2_Te_3_ notably resemble a continuum of transitions, similar to those observed in 3D and 2D materials, which give rise to a continuous variation with some spectral maxima compared to the elemental chalcogen chains. In particular, 3D bulk Bi_2_Te_3_ has recently gained a wide interest due to its topological features^85^ and its successful 2D exfoliation.^64^

These materials exhibit lower QP band gaps, exciton binding energies (<1 eV) and peak intensities. In the As_2_S_3_ spectrum, two main peaks, composed of several excitations, appear below the direct electronic gap (3.04 eV), as shown in Figure 7a and highlighted in the related inset. The first of these peaks is composed of three bright excitations, occurring at 2.71, 2.75, and 2.79 eV, falling within the blue-violet region of the visible spectrum and exhibiting small binding energies of 0.33, 0.29, and 0.25 eV, respectively, if measured with respect to the QP gap. Being so close in energy, these excitons originate from the same single-particle transitions, mainly involving the first two degenerate occupied bands and the first six degenerate unoccupied bands, concentrated around Γ. These band pair contributions are shown in detail in Figure 7b,c and in S13a in the Supporting Information. The excitonic wave function of the lowest bound state is depicted in Figure 6c and presents an estimated radius of 14.4 Å. Similarly, the second more intense peak arises from two nearly degenerate excitations occurring at 3.00 and 3.02 eV. As shown in Figure 13b,c in the Supporting Information, they stem from transitions between the first two degenerate occupied bands and the first and the fourth degenerate unoccupied bands, concentrated around Γ. To conclude the discussion, it is worth mentioning that, in the case of As_2_S_3_, the ev*GW* calculations dramatically modify the excitonic spectrum. In particular, the self-consistent procedure causes the contributions to the excitons to migrate from the region around *Z* (see Figure 7a–c in the Supporting Information) to that around Γ, as previously shown. The main effect of this redistribution is a drastic and anomalous drop of the exciton binding energy of the lowest bright exciton from 0.89 to 0.33 eV, with a related increase of the exciton radius from 12.0 to 14.4 Å.

**Figure 7:**
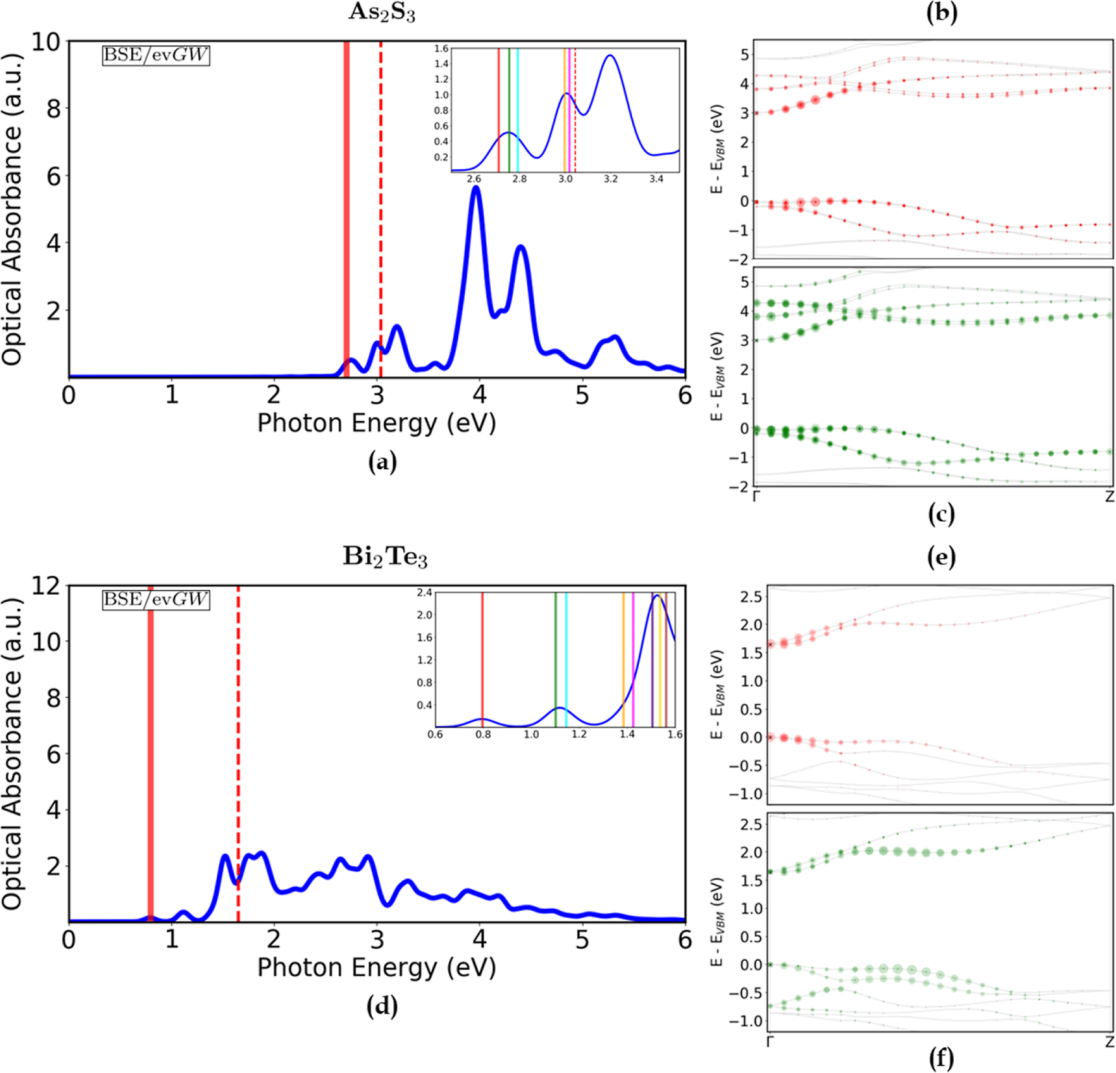
[Left] Absorption spectra (solid blue) of (**a**) As_2_S_3_ and (**d**) Bi_2_Te_3_, expressed in terms of the optical absorbance *A*(ω), calculated at the ev*GW*/BSE level. The corresponding ev*GW*–corrected direct electronic band gaps (dashed red) are shown as a reference. A broadening of 50 meV was used. [Right] Electronic band structures (solid gray), calculated at the ev*GW* level, of As_2_S_3_ (**b**,**c**) and Bi_2_Te_3_ (**e**,**f**). The colored dots (red and green) represent the single-particle transitions contributing to the first two bright excitons, and their size is proportional to the intensity of the transition - renormalized to the highest value. The corresponding excitonic peaks are highlighted in the relative absorption spectra (solid red and green) and in their insets, together with other meaningful higher bound states below the electronic gap (see Figures S13 and S14 in the Supporting Information). Energy zero is set as the top of the valence bands. SOC and semicore corrections were included.

Finally, in the Bi_2_Te_3_ case (Figure 7d), the first exciton peak below the gap (1.65 eV) occurs at 0.8 eV, thus in the near-IR region. This gives an exciton binding energy of 0.85 eV, which is again significantly lower than that of the other three materials. This value represents a dramatic enhancement compared to bulk Bi_2_Te_3_, where exciton binding energies are only on the order of tens of meV — 14 meV experimentally^86^ and about 30 meV theoretically^87^ — underscoring the crucial role of reduced dimensionality and dielectric confinement in electron–hole interactions.

The corresponding excitonic wave function of the lowest bright exciton (Figure 6d) has a radius of 13.2 Å. This first excitation, as well as the others with higher energies below the gap, mainly arises from transitions involving the two highest degenerate valence bands and the two lowest degenerate conduction bands close to Γ (see Figure 7e). The second peak, at 1.1 eV, is composed of two excitations, whose transitions stem from the same bands (see Figures 7f and S14a in the Supporting Information) in the region between Γ and 1/2 Γ*Z*. These first two peaks have a very low intensity. The last peak below the gap is indeed the most intense and it is composed of three nearly degenerate excitations (see inset in Figure 7d), located at 1.51, 1.54, and 1.56 eV. These excitons are composed of a bigger number of bands and a broader energy range, as shown in Figure S14e–g in the Supporting Information. To complement the optical characterization, we estimated the radiative lifetimes of the lowest bright excitons following the formalism described in refs 30,88 , particularly the zero-momentum lifetimes τ(0) and their thermally averaged effective values τ_eff_ obtained at 300 K. We find that τ(0) increases from the elemental chains S_3_ (2.4 ps) and Te_3_ (9.5 ps) to the related compounds As_2_S_3_ (36 ps) and Bi_2_Te_3_ (0.11 ns). The corresponding effective radiative lifetimes τ_eff_ get enhanced with respect to the intrinsic radiative lifetimes τ(0) by *a* factor ≈100 at room temperature and follow the same trend, ranging from 80 ps (S_3_) and 0.52 ns (Te_3_) to 1.1 ns (As_2_S_3_) and 9.7 ns (Bi_2_Te_3_). These values are comparable to those reported for strongly bound excitons in CNTs by Spataru et al.,^30^ about 10 ps for τ(0) and 1–2 ns for τ_eff_. These values are also comparable to some 2D semiconductors such as monolayer MoS_2_, MoSe_2_, WS_2_ and WSe_2_ ^89^ and tellurene.^83^

In summary, in the absorption spectra of the four 1D materials, enhanced excitonic effects are visible, in particular, strongly bright bound excitons. For the freestanding quantum wires under consideration, the low screening, due to the 1D electronic system, plays a prominent role. Due to the reduced dimensionality, electrons and holes experience stronger Coulomb interactions, as screening is less effective in 1D compared to bulk systems, as highlighted by the comparison of the exciton binding energies of Te_3_ and Bi_2_Te_3_. This leads to the formation of tightly bound excitons with large binding energies, significantly influencing the optical properties, showing that these exfoliable atomic chains reach an extreme regime of quantum confinement and Coulomb interaction, surpassing all previously reported (quasi-)­1D systems with larger dimensions. For instance, conjugated polymer chains such as poly para-phenylenevinylene (PPV) exhibit binding energies of 0.6–0.7 eV,^30^ while single-walled CNTs show values ranging from 0.1 to 1 eV depending on the tube diameter.^90^ Even in semiconductor–insulator quantum wires of GaAs, CdSe, and InP with diameters of 4–6 nm, the binding energies remain in the range of 120–260 meV.^91^

### Understanding 1D Excitons

As shown in Table 2, the lowest-energy excitons exhibit large binding energies on the order of 1–2 eV (except for the case of As_2_S_3_). These strong excitonic effects can be understood through their electronic structures (Figures 5 and 7) and the resulting electronic screening.

Since the two-particle states primarily arise from the lowest conduction and highest valence bands near *Z* for S_3_ and Te_3_ (or near Γ for As_2_S_3_ and Bi_2_Te_3_), we employ a two-band model with the effective-mass approximation (EMA) centered around these high-symmetry points. To this end, we make use of the electronic band structures calculated at the DFT level (reported in Figure S5 in the Supporting Information) with SOC included to extract effective masses of the electrons and holes forming the lowest exciton bound state. The effective masses were obtained from parabolic fits of the valence and conduction bands extrema near the Γ or *Z* points, depending on the system.

The internal motion of the bound electron–hole pair along the wire axis *z* is described, within the EMA, by the Schrödinger equation

1 $$\left\{-\frac{\hbar^{2}}{2\mu }\frac{d^{2}}{dz^{2}}+W\left(z\right)\right\}\phi \left(z\right)=-E_{b}^{M}\phi \left(z\right)$$

where *W*(*z*) represents the attractive screened Coulomb interaction. The wave function ϕ­(*z*) describes the internal motion of the lowest exciton with binding energy *E*_b_^M^. The kinetic energy is ruled by μ, i.e. the reduced effective mass of the electron–hole pair, taking values μ = 0.39, 0.30, 0.16, and 0.10, in units of the electron mass, for S_3_, Te_3_, As_2_S_3_, and Bi_2_Te_3_, respectively.

The screened potential *W*(*z*) is derived from the Poisson equation, modeling the quantum wire as a cylinder with radius *R* and a static 1D electronic polarizability α_1D_^0^ = *S*(ϵ_1_(0)-1)/4π calculated within the independent particle picture using DFT eigenvalues and eigenfunctions, in a similar fashion as in the 2D case.^92^ Here, ϵ_1_(0) is the real part of the supercell dielectric function at ω = 0 for light polarized in the *z* direction, and *S* is the xy area of the supercell.We obtain the polarizabilities α_1D_^0^ = 12.06 (S_3_), 17.43 (Te_3_), 26.05 (As_2_S_3_), and 51.63 (Bi_2_Te_3_) Å^2^. The Fourier transform of *W*(*z*) is given by^93^

2 $$\mathrm{W̃}\left(q\right)=-e^{2}\frac{{Ṽ}_{bare}\left(q\right)}{1+\alpha_{1D}^{0}q^{2}{Ṽ}_{bare}\left(q\right)}$$

where ${Ṽ}_{bare}\left(q\right)$ is the Fourier-transformed bare Coulomb potential, averaged over the wire cross-section using different distribution functions *f*(ρ). Because of the long-range interaction between charged particles, only small wave vectors *q* play a role. Therefore, a possible modification of the denominator by the nonlocality of the screening reaction is omitted. We assume a screening reaction that is localized at the rod surface with $f\left(\rho \right)=\frac{1}{2\pi R}\delta \left(\rho -R\right)$ or homogeneously distributed over the wire cross-section with $f\left(\rho \right)=\frac{1}{\pi R^{2}}\theta \left(R-\rho \right)$ . The resulting bare potentials are

3 $${Ṽ}_{bare}\left(q\right)=2K_{0}\left(\left|q\right|R\right)$$

4 $${Ṽ}_{bare}\left(q\right)=\frac{4}{q^{2}R^{2}}\left[1-\left|q\right|RK_{1}\left(\left|q\right|R\right)\right]$$

with zeroth- (*K*_0_) and first-order (*K*_1_) modified Bessel functions. In real space, they become, respectively, the softcore and modified softcore 1D Coulomb potentials^94^

5 $$V_{bare}\left(z\right)=\frac{1}{\sqrt{z^{2}+R^{2}}}$$

6 $$V_{bare}\left(z\right)=\frac{2}{R^{2}}\left[\sqrt{R^{2}+z^{2}}-\left|z\right|\right]$$

The influence of the substrate or environment on the screening in the wires can be described by averaging its dielectric constant with that of the vacuum, similar to the approach used in 2D systems.^95^ The complexity of the screened potential (2) forbids an analytic solution of the exciton problem ([Disp-formula eq1]). Therefore, we apply a variational treatment with the trial function.

7 $$\phi \left(z\right)=\sqrt{\frac{2s^{3}}{a_{exc}^{3}}}\left|z\right|e^{-s\left|z\right|/a_{exc}}$$

where *s* is a variational parameter and *a*_exc_ = *a*_B_ *m*_e_/μ is the effective Bohr radius. In the limit *s* = 1, (7) represents the ground-state wave function of the 1D hydrogen atom with the bare potential $W\left(z\right)=-\frac{e^{2}}{\left|z\right|}$ .^96^ From ([Disp-formula eq1]), ([Disp-formula eq2]), and ([Disp-formula eq7]), we obtain the variational expression

8 $$E_{b}^{M}\left(s\right)=R_{exc}\left[-s^{2}+s\frac{4}{\pi }\int_{0}^{\infty }dt\frac{1-3t^{2}}{{\left(1+t^{2}\right)}^{3}}\left(-\frac{1}{e^{2}}\right)\mathrm{W̃}\left(\frac{2s}{a_{exc}}t\right)\right]$$

with *R*_exc_ = *R*_H_ μ/*m*_e_ and *R*_H_ = 1 Ry. Its maximum at *s* = *s*_0_, for which $\frac{d}{ds}E_{b}^{M}\left(s\right){|}_{s=s_{0}}=0$ , yields the exciton binding energies *E*_b_^M^ and exciton radii *r*_b_^M^, listed in Table 2. The exciton radii, derived from the BSE calculations and from the effective-mass model, are several times larger than the lattice constant, suggesting the Wannier–Mott-like nature of these bound states.

The estimated binding energies vary with the softcore or modified softcore 1D hydrogen potential used by less than 20%. This effect is reduced for the average electron–hole distances *r*_b_^M^. Consequently, one can conclude that the distribution of interaction of the charged particles over the wire cross-section is of minor influence. More important is the screening itself. Despite the small wire radius *R*, the screening significantly reduces the value *E*_b_^M^ with respect to *R*_exc_ by *a* factor of 2–3. In the case of the excitonic radii *r*_b_^M^, the screening effect is somewhat enhanced. The screening reaction of the electron gas in the considered 1D systems is of extreme importance to understand the electron–hole attraction. Indeed, the reaction can be described by a 1D electronic polarizability as indicated in formula [Disp-formula eq2].

The comparison of the model estimates *E*_b_^M^ and *r*_b_^M^ with the corresponding ab initio values *E*_b_^BSE/evGW^ (or $E_{b}^{{BSE/G}_{0}W_{0}}$ ) and *r*_b_^BSE/evGW^ (or $r_{b}^{{BSE/G}_{0}W_{0}}$ ), reported in Table 2, shows the same chemical trends along the row S_3_, Te_3_, As_2_S_3_, and Bi_2_Te_3_, but also very similar values. This holds especially for the monatomic chains S_3_ and Te_3_, where the binding energies agree very well, applying the correspondence BSE/G_0_W_0_ ↔ softcore potential and BSE/evGW ↔ modified softcore potential. In the case of the more complex systems As_2_S_3_ and Bi_2_Te_3_, the quantitative agreement is reduced, but the same order of magnitude is still guaranteed. In any case, we confirm that the exciton binding can be reliably predicted by the model based on a parabolic two-band model, generalized 1D hydrogen Coulomb potentials, and a screening reaction of the 1D electron gas described by means of a static electronic polarizability.

## Conclusions

We have presented a first-principles investigation of a class of potentially exfoliable atomic wires identified through HT screening. We explored the structural, energetic, vibrational, electronic, and optical properties of four promising chain-like materials — S_3_, Te_3_, As_2_S_3_, and Bi_2_Te_3_ — using DFT, DFPT, and MBPT, including *GW* corrections and BSE calculations.

The selected chain systems are dynamically stable, with the exception of As_2_S_3_, where the lowest TA phonon branch exhibits a slight negative dispersion, suggesting a tendency to relax into a larger unit cell at 0 K. The phonon dispersions display the characteristic twisting acoustic mode of realistic 1D materials. Interestingly, the IR spectra of the homopolar S_3_ and Te_3_ chains exhibit one weak absorption peak where the dipole moment arises from the geometrical conformation rather than from differences in electronegativity. In contrast, As_2_S_3_ and Bi_2_Te_3_, with partly ionic bonding, display, as expected, more IR-active phonons and significantly stronger absorption peaks.

The chain materials exhibit unique band structures with flat bands and substantial band gap separations, indicating potential for exotic electronic phenomena related to electrons and/or holes with relatively heavy effective masses. Because of the weak electronic screening, the band structures are significantly influenced by single-particle QP effects, at least in Hedin’s *GW* approximation. The gaps and interband distances are greatly increased compared to the KS values from the DFT framework. The gaps are opened by 1–2.8 eV in the first *G*_0_ *W*_0_ iteration step. Further openings have been observed in the limit of converged *GW* calculations, at least with respect to the single-particle eigenvalues. Interestingly, the QP effects also influence the band dispersion. While the KS band structures indicate indirect semiconductors with the maxima of the uppermost valence band on the Γ*Z* line, the self-consistent QP treatment tends to indirect-to-direct transitions with direct a gap at *Z* in the homopolar S_3_ and Te_3_ chains and at Γ in the polar 1D materials As_2_S_3_ and Bi_2_Te_3_.

The most relevant many-body features appear in the optical spectra. Besides the QP blueshift of the absorption threshold, a significant redistribution of the spectral strengths occurs due to the electron–hole attraction, the excitonic effects. Peaks of bound excitons appear below the single-particle absorption edge, defined by the QP gap. The homopolar chains S_3_ and Te_3_ show significant exciton binding energies, indicative of highly localized excitonic states, while the ionic compounds As_2_S_3_ and Bi_2_Te_3_ exhibit a continuum of transitions with smaller binding energies.

The extremely strong excitonic effects are explained by introducing a model based on the effective mass approximation of the kinetic energy of the internal electron–hole motion and a screened Coulomb potential in 1D systems. The latter describes the screening by a 1D static electronic polarizability of the electronic gaps in the wires. Indeed, the large exciton binding energies, of the order of 0.8–2.3 eV, can be explained by the small electronic polarizabilities and the large interband masses of 0.1–0.7 m_e_, at least for freestanding chains.

Given these extreme properties, S_3_ and Te_3_ are promising candidates for optoelectronic applications, particularly in UV- and visible-absorption devices due to their strong excitonic effects. In the case of the polar chain materials, additional strong absorption appears in the range of optical phonons. The lower exciton binding energies and broader absorption spectra of As_2_S_3_ and Bi_2_Te_3_ make them suitable for photovoltaic and IR applications. These findings suggest that exfoliable 1D wires could be key materials for future nanoscale electronic and optoelectronic technologies.

## Methods

The calculation of ground-state properties within the framework of DFT is carried out using the Quantum ESPRESSO (QE) distribution.^97,98^ Phonon dispersions are computed using density-functional perturbation theory (DFPT),^75^ using **q**-grids of 1 × 1 × 6 (Te_3_), 1 × 1 × 10 (S_3_ and Bi_2_Te_3_) and 1 × 1 × 20 (As_2_S_3_). Pseudopotentials are taken from the SSSP library,^99^ v1.1 PBE efficiency, using the suggested kinetic energy cutoffs, and a minimum vacuum distance of 21 Å (up to a maximum of 27 Å). The correct long-wavelength behavior of the acoustic phonons is imposed using the acoustic sum rule that includes rotational symmetries, implemented as described in ref 71. The IR spectra are obtained from the dynmat postprocessing module of QE, starting from the eigenmodes and Born effective charges previously computed. The intensities of the peaks are smeared with a Gaussian function of half-width 9 cm^–1^, and normalized by the volume of the wires (4π/*V*_0_), which we define as the quantum volume^100^ calculated using the Quantum ENVIRON package.^101^

Concerning electronic properties, a norm-conserving, fully relativistic pseudopotential from the PseudoDojo repository (v0.4)^102^ is employed to account for spin–orbit coupling (SOC) and include semicore electrons, using an exchange–correlation functional within the generalized gradient approximation (GGA) according to Perdew, Burke and Ernzerhof (PBE).^103^ Kinetic energy cutoffs of 110 (S_3_), 70 (Te_3_), 80 (As_2_S_3_) and 100 (Bi_2_Te_3_) Ry are chosen. A uniform Monkhorst–Pack^104^ **k**-point mesh, with a dimension of 1 × 1 × 12, is employed. To prevent interaction between periodic replicas, a minimum vacuum region of 16 Å along the nonperiodic (*xy*) directions is introduced. Structural relaxation is considered as converged when the maximum component of the residual ionic forces dropped below 10^–8^ Ry/Bohr.

From the DFT eigenvalues and eigenvectors obtained, MBPT calculations are carried out using the Yambo code,^105,106^ specifically employing the *G*_0_ *W*_0_ and eigenvalue self-consistent *GW* (ev*GW*) methods for the quasiparticle corrections of the electronic states and the BSE to account for the *e-h* interaction in the excited states, e.g., in the optical absorption spectra.^107–110^ The BSE was solved within the Tamm–Dancoff approximation (TDA). While the TDA is known to fail for low-dimensional systems,^111^ it is still reliable for absorption spectra when the perturbing field is polarized along the wire axis. For *GW* calculations, cutoffs of 240, 280, 340, and 240 Ry were used to calculate the difference between the exchange part of the electron self-energy Σ_x_ and the XC functional *V*_xc_, respectively for S_3_, Te_3_, As_2_S_3_ and Bi_2_Te_3_, while 12 Ry are used for the correlation part of the self-energy Σ_c_. Additionally, 1582 (S_3_), 1402 (Te_3_), 1452 (As_2_S_3_) and 622 (Bi_2_Te_3_) empty bands are included in the calculation of the Σ_c_ [Fn fn3]. A cylindrical cutoff to the Coulomb potential along the nonperiodic directions (*xy*) is also used, as implemented in the Yambo code,^106^ to avoid artificial interaction between a wire and its replica. *GW* calculations are performed using the plasmon-pole approximation (PPA) to model the frequency dependence of the dielectric function, as implemented in the Yambo code. For the BSE Hamiltonian, a total of 10 occupied states and 10 unoccupied states are employed. The convergence with respect to the **k**-points for both the QP corrections and the BSE is carefully checked, a **k**-point grid of 1 × 1 × 48 has been used.

Grillo, S.; Cignarella, C.; Bechstedt, F.; Gori, P.; Palummo, M.; Campi, D.; Marzari, N.; Pulci, O. Quasiparticle effects and strong excitonic features in exfoliable 1D semiconducting materials. 2025, arXiv:2510.09194. arXiv. https://arxiv.org/abs/2510.09194 (accessed October 13, 2025).

## Supplementary Material



## Footnotes

a The nomenclature used corresponds to the number of atoms of each atomic species in the unit cell.

b A slight frequency misalignment in Bi_2_Te_3_ between IR and phonon dispersions arises from applying different ASRs during postprocessing, since the same 1D-ASRs with full Born-Huang conditions^71^ are not implemented in the “dynmat” module.

c Convergence with respect to empty states has been proven to be a critical factor in 1D systems. For further details, we refer the reader to the Supporting Information.

## Notes

Discussion on the dependence of the empty states on the cell vacuum and results for S_3_. Electronic band structures at the DFT level of theory for the four nanowires, with and without SOC. Absorption spectra and electronic band stuctures at the G_0_W_0_/BSE level and electronic band stuctures at the evGW level. Detailed table summarizing the MBPT results (PDF).
