# Supplementary material for: Quasiparticle Effects and Strong Excitonic Features in Exfoliable 1D Semiconducting Materials
Source: ACS Nano. 2026 Jan 13;20(3):2664–77. doi: 10.1021/acsnano.5c14061 (PMC12854755; doi:10.1021/acsnano.5c14061)
Supplement: Supplementary file 1 [file nn5c14061_si_001.pdf]

# Supporting Information to: Quasiparticle effects and strong excitonic features in exfoliable 1D semiconducting materials

Simone Grillo,[*,†,‡] Chiara Cignarella,[*,¶,§] Friedhelm Bechstedt,[‖] Paola Gori,[⊥] Maurizia Palummo,[†] Davide Campi,[#,@] Nicola Marzari,[¶,△,§] and Olivia Pulci[*,†]

† *Dipartimento di Fisica, Università di Roma Tor Vergata and INFN, 00133 Rome, Italy*

‡*Max Planck Institute for the Structure and Dynamics of Matter (MPSD) and Center for Free-Electron Laser Science (CFEL), 22761 Hamburg, Germany*

¶*Theory and Simulation of Materials (THEOS), and National Centre for Computational Design and Discovery of Novel Materials (MARVEL), École Polytechnique Fédérale de Lausanne, 1015 Lausanne, Switzerland*

§*U Bremen Excellence Chair, Bremen Center for Computational Materials Science, and MAPEX Center for Materials and Processes, University of Bremen, 28359 Bremen, Germany*

‖*Institut für Festkörpertheorie und -optik, Friedrich-Schiller-Universität Jena, 07743 Jena, Germany*

⊥*Dipartimento di Ingegneria Industriale, Elettronica, e Meccanica, Università Roma Tre, 00154 Roma, Italy*

#*Dipartimento di Scienza dei Materiali, Università degli studi di Milano Bicocca, 20126 Milan, Italy*

@*Bicocca Quantum Technologies (BiQuTe) Centre, I-20126 Milan, Italy*

△*PSI Center for Scientific Computing, Theory and Data, 5232 Villigen, Switzerland*

E-mail: simone.grillo@roma2.infn.it; ccignare@uni-bremen.de; olivia.pulci@roma2.infn.it

The Supporting Information contains:

- discussion of the dependence of the empty states on the cell vacuum (Section *Dependence of the empty states on the cell vacuum*).
- the electronic band structures at DFT-PBE level of theory, with and without SOC (Section *DFT electronic structures*);
- supporting analysis for the four wires at the MBPT level of theory: *Absorption spectra and electronic band structures at the $G_0W_0$ and $G_0W_0$/BSE level* and *Electronic band structures at the evGW level.* We provide in Table 1 a detailed summary of the results, at both the $G_0W_0$ and evGW level.

## Dependence of the empty states on the cell vacuum

DFT calculations of periodic low-dimensional materials such as nanowires, 2D materials, and thin films, require the inclusion of a vacuum region for achieving accurate results. These materials are often simulated using periodic boundary conditions, which replicate the unit cell throughout space. For low-dimensional systems, these periodic replicas can cause unintended interactions between the material and its periodic images in the non-periodic directions. By including a sufficiently large vacuum region around the material, typically in the range of 15-20 Å, one tries to minimize these spurious interactions, particularly vdW forces and electrostatic interactions. The inclusion of vacuum is therefore a fundamental step in DFT simulations of low-dimensional materials to ensure the physical relevance and reliability of the calculated properties.

In recent years, researchers have been developing advanced techniques within DFT to address the limitations of the traditional approach of including large vacuum regions around low-dimensional materials. While adding vacuum is effective in reducing short-range interactions between periodic images, it significantly increases the computational cost, especially for large

supercells. This is of special importance for MBPT treatment of the excited electronic states with long-range Coulomb interactions. To make these calculations more efficient, efforts have been directed toward implementing Coulomb cutoff methods. Coulomb cutoffs are designed to selectively limit the range of the Coulomb interaction in certain directions, effectively reducing the artificial electrostatic interactions between periodic images without the need for extensive vacuum regions. By truncating the Coulomb potential, these methods ensure that the interaction decays rapidly beyond a certain distance, which allows for the use of smaller unit cells and less vacuum. This reduction not only decreases computational resources but also improves the accuracy of simulations by focusing on the intrinsic properties of the material itself rather than the vacuum-induced artifacts.

In particular, two Coulomb cutoff techniques were implemented in Quantum ESPRESSO for both 1D[1] and 2D[2] systems. These methods have proven to be highly effective in accelerating the convergence of ground-state properties — such as the total energy, the Fermi level, and the phonon dispersion — without the need for excessively large vacuum regions. However they are less effective in the calculation of the energy dispersion, particularly for empty states. This limitation is especially pronounced in 1D systems, where achieving convergence of the band structure with respect to the vacuum region is virtually impossible.

Here, we present an example based on of $S_3$, one of the systems proposed in our manuscript. In Fig. 1 we present the band structure of $S_3$, calculated with two different vacuum regions: 16 Å (cyan) and 18 Å (blue). Even with a small increase of just 2 Å, the electronic bands are well converged only up to few eV above the Fermi level (red dashed line).

This difficulty can be attributed to the reduced dimensionality of 1D systems. In these systems, electronic screening is significantly less effective compared to 2D or bulk materials, leading to stronger Coulomb interactions. As a result, the electron wavefunctions become highly delocalized into the vacuum region, making it challenging to isolate the system's intrinsic properties. This issue significantly hinders the calculation of excited-state properties in 1D systems. Indeed, most computational codes available for simulating such properties in

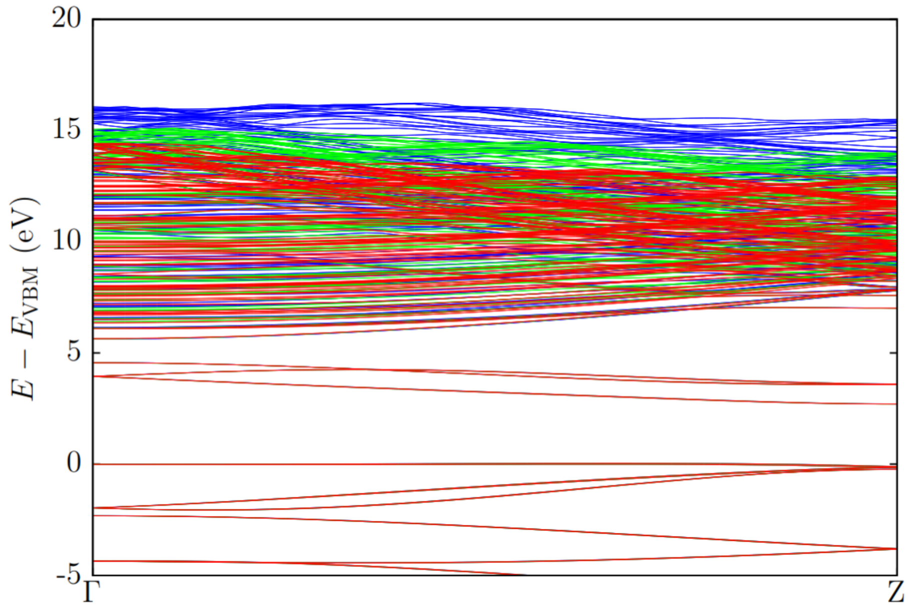


Figure 1: Calculated band structure, at the DFT level, of the $S_3$ wire using a vacuum of 16 Å (blue), 18 Å (green) and 22 Å (red) in the non-periodic directions. The electronic bands are well converged only up to few eV above the energy zero, set as the top of the valence bands.

periodic materials rely on summations over empty states to compute key quantities like the correlation self-energy $\Sigma_c$. Therefore, the inability to properly converge the band structure of 1D materials due to insufficient vacuum can lead to inaccuracies in predicting excited-state phenomena. To bypass the issue, we employed the following strategy:

- The QP corrections to the band gap were calculated simultaneously for varying amounts of vacuum.
- For each vacuum level, the summations over empty states were truncated at an increasing energy threshold.

The reference for the zero energy should be chosen accordingly, preferably starting from the Fermi level. An example of this procedure is shown in Fig. 2. As the vacuum increases, the number of empty states within each energy threshold also increases, leading to a significantly higher computational cost at each step. Our calculations indicate that a vacuum of 16 Å and an energy threshold of 35 eV (from the Fermi level) are generally sufficient to obtain

an acceptable convergence of the band gap within less than 20 meV, with a discrepancy of about 10 meV compared to a vacuum distance of 22 Å or more. This "recipe" allows for simultaneous control of the convergence with respect to both vacuum and empty states in MBPT calculations, with the QP correction to the band gap serving as the convergence parameter, bypassing the direct dependence of the results on these quantities while reducing the computational cost.

In Fig. 3, we provide an example of additional convergence (standard) procedures used for the MBPT calculations, including $GW$ and BSE, for $S_3$.

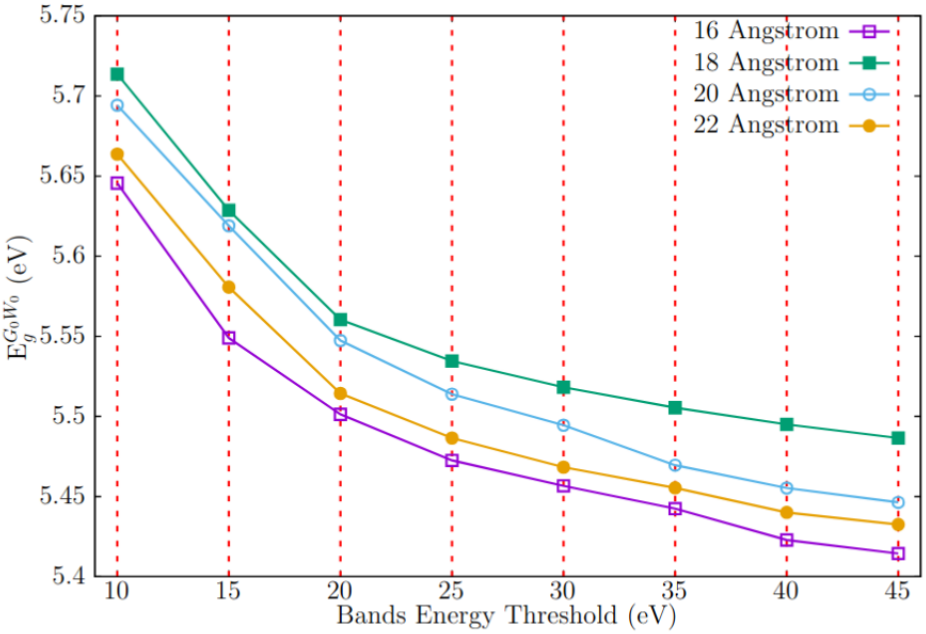


Figure 2: Convergence of the $G_0W_0$-corrected band gap of $S_3$ with respect to the energy threshold for empty bands included in the summations. The different curves represent the different vacuum used in the calculations. A vacuum of 16 Å and an energy threshold of 20 eV (from the Fermi level) are generally sufficient to converge the correction to the band gap, resulting in a discrepancy of less than 1% compared to a vacuum of up to 22 Å.

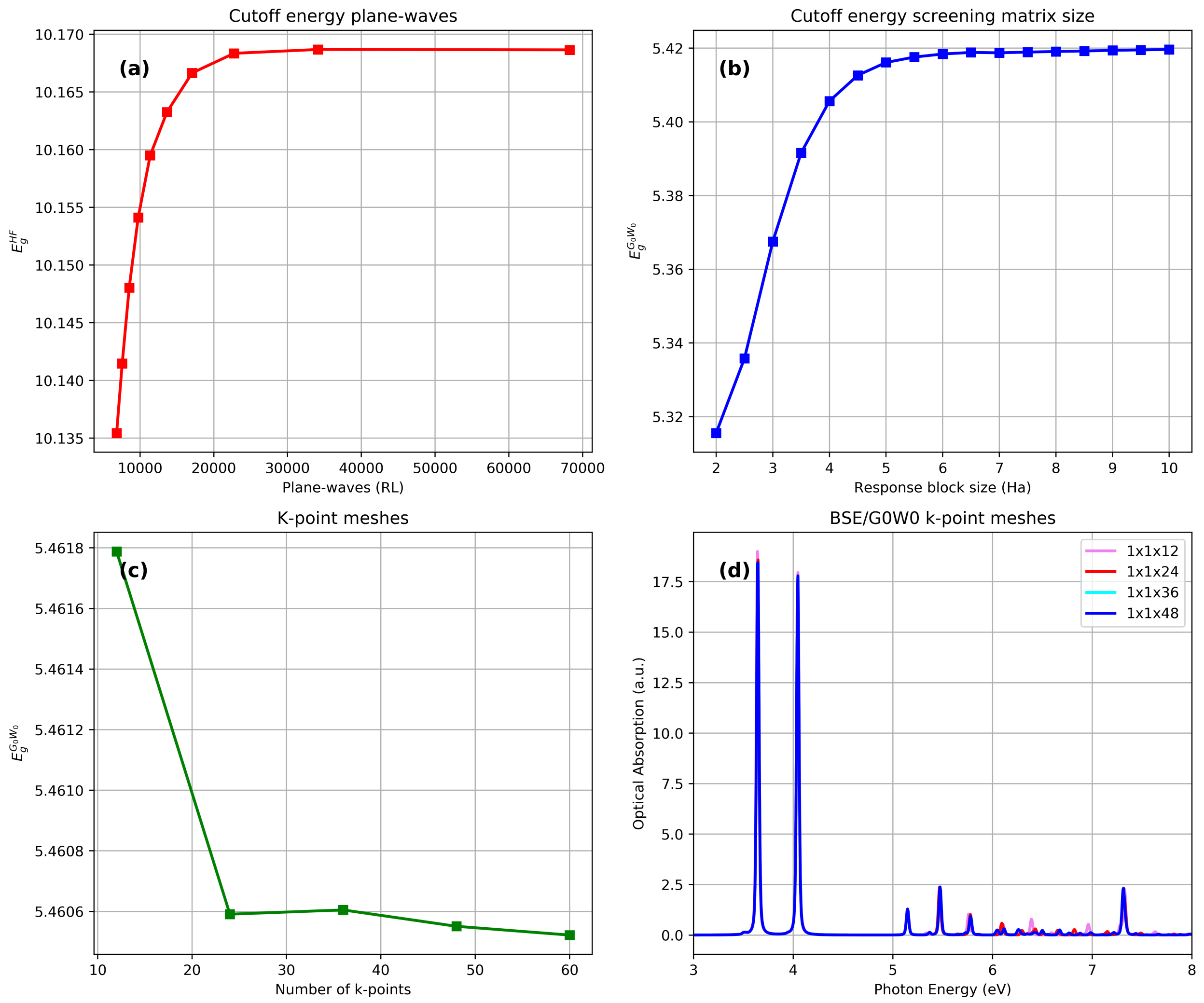


Figure 3: (a) Convergence of the HF-corrected band gap of $S_3$ with respect to the number of plane-waves. (b) Convergence of the $G_0W_0$-corrected band gap of $S_3$ with respect to the size of the screening matrix. (c) Convergence of the $G_0W_0$-corrected band gap of $S_3$ with respect to the k-point mesh. (d) Convergence of the BSE/$G_0W_0$ spectrum with respect to the k-point mesh.

## DFT electronic structures

In this section we present the electronic band structures of the wires at DFT-PBE level without and with the inclusion of SOC, respectively, in Figs. 4-5. We limit the detailed discussion on the SOC case. As pointed out in the main text, all four wires possess an indirect-gap nature, often presenting a small difference between the direct and indirect electronic gap. The corresponding values are listed in Table 1 in the main text.

$S_3$ has an estimated DFT indirect electronic gap of 2.66 eV (Fig. 4a), with a direct gap of 2.76 eV. The CBM is located at the high-symmetry Z point, while the VBM is located between the Γ and Z points of the SOC-split flat valence band. The band structure of $Te_3$ shows many similarities with that of $S_3$, with an estimated indirect electronic gap of 1.43 eV (Fig. 4b). Both the VBM and the CBM are located near the high-symmetry Z point. The direct electronic gap is only 40 meV greater and it is located close to the Z point. $As_2S_3$ and $Bi_2Te_3$ exhibit similar structures and band structures, and both are significantly influenced by SOC. $As_2S_3$ possesses an indirect band gap of 1.16 eV (Fig. 4c), while the direct gap was estimated to be 1.26 eV. In this material, the CBM is located at the Γ point, while the VBM is situated between the Γ and Z points. In contrast, $Bi_2Te_3$ is a nearly-direct narrow-gap semiconductor with an electronic band gap of 0.419 eV (Fig. 4d). Here, the VBM is located at the Γ point, and the CBM is very close to the Γ point. Indeed, the direct band gap at Γ is only 2 meV larger than the indirect band gap.

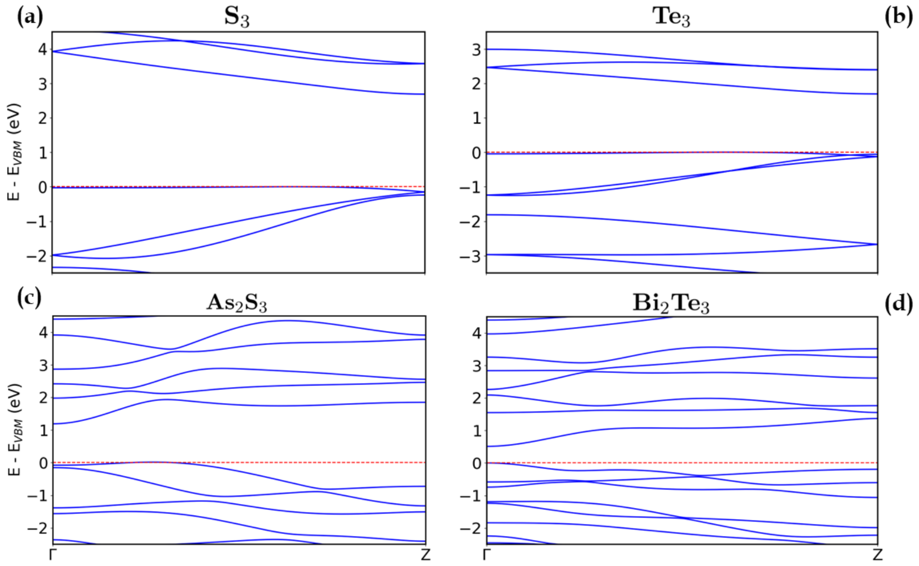


Figure 4: Electronic band structures of (**a**) $S_3$, (**b**) $Te_3$, (**c**) $As_2S_3$ and (**d**) $Bi_2Te_3$, calculated at the DFT level, using a GGA-PBE XC functional. Energy zero is set as the top of the valence bands. The zero level is highlighted with a red dotted line.

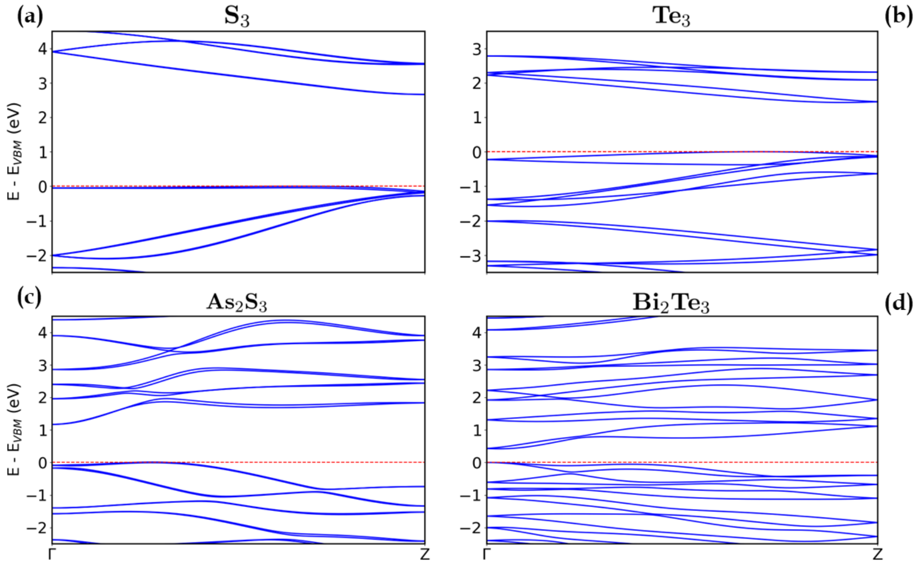


Figure 5: Electronic band structures of (**a**) $S_3$, (**b**) $Te_3$, (**c**) $As_2S_3$ and (**d**) $Bi_2Te_3$, calculated at the DFT level, using a GGA-PBE XC functional. SOC was included. Energy zero is set as the top of the valence bands. The zero level is highlighted with a red dotted line.

# MBPT supporting data

Table 1: Calculated lowest direct electronic band gaps ($E_g$), at the $G_0W_0$ and ev$GW$ level, together with the BSE optical gaps ($E_{opt}^{\mathrm{BSE}}$) and the corresponding binding energies ($E_b^{\mathrm{BSE}}$) and radii ($r_b^{\mathrm{BSE}}$) of the lowest bright excitons. In the case of an indirect band gap, the corresponding direct band gap is also reported in square brackets. The reported values are expressed in eV, except for the radius which is expressed in Å. SOC and semi-core corrections were included.

| | $E_g^{G_0W_0}$ | $E_{opt}^{\mathrm{BSE}/G_0W_0}$ | $E_b^{\mathrm{BSE}/G_0W_0}$ | $r_b^{\mathrm{BSE}/G_0W_0}$ | $E_g^{\mathrm{ev}GW}$ | $E_{opt}^{\mathrm{BSE/ev}GW}$ | $E_b^{\mathrm{BSE/ev}GW}$ | $r_b^{\mathrm{BSE/ev}GW}$ |
|---|---|---|---|---|---|---|---|---|
| **$S_3$** | 5.46 | 3.62 | 1.93 | 12.5 | 6.25 | 4.07 | 2.27 | 12.4 |
| | [5.55] | | | | [6.34] | | | |
| **$Te_3$** | 3.47 | 1.85 | 1.65 | 14.8 | 4.27 | 2.17 | 2.14 | 14.7 |
| | [3.50] | | | | [4.31] | | | |
| **$As_2S_3$** | 2.26 | 1.41 | 0.89 | 12.00 | 3.00 | 2.71 | 0.33 | 14.4 |
| | [2.30] | | | | [3.04] | | | |
| **$Bi_2Te_3$** | 1.54 | 0.77 | 0.77 | 13.2 | 1.65 | 0.80 | 0.85 | 13.2 |

**Absorption spectra and electronic band structures at the $G_0W_0$ and $G_0W_0$/BSE level**

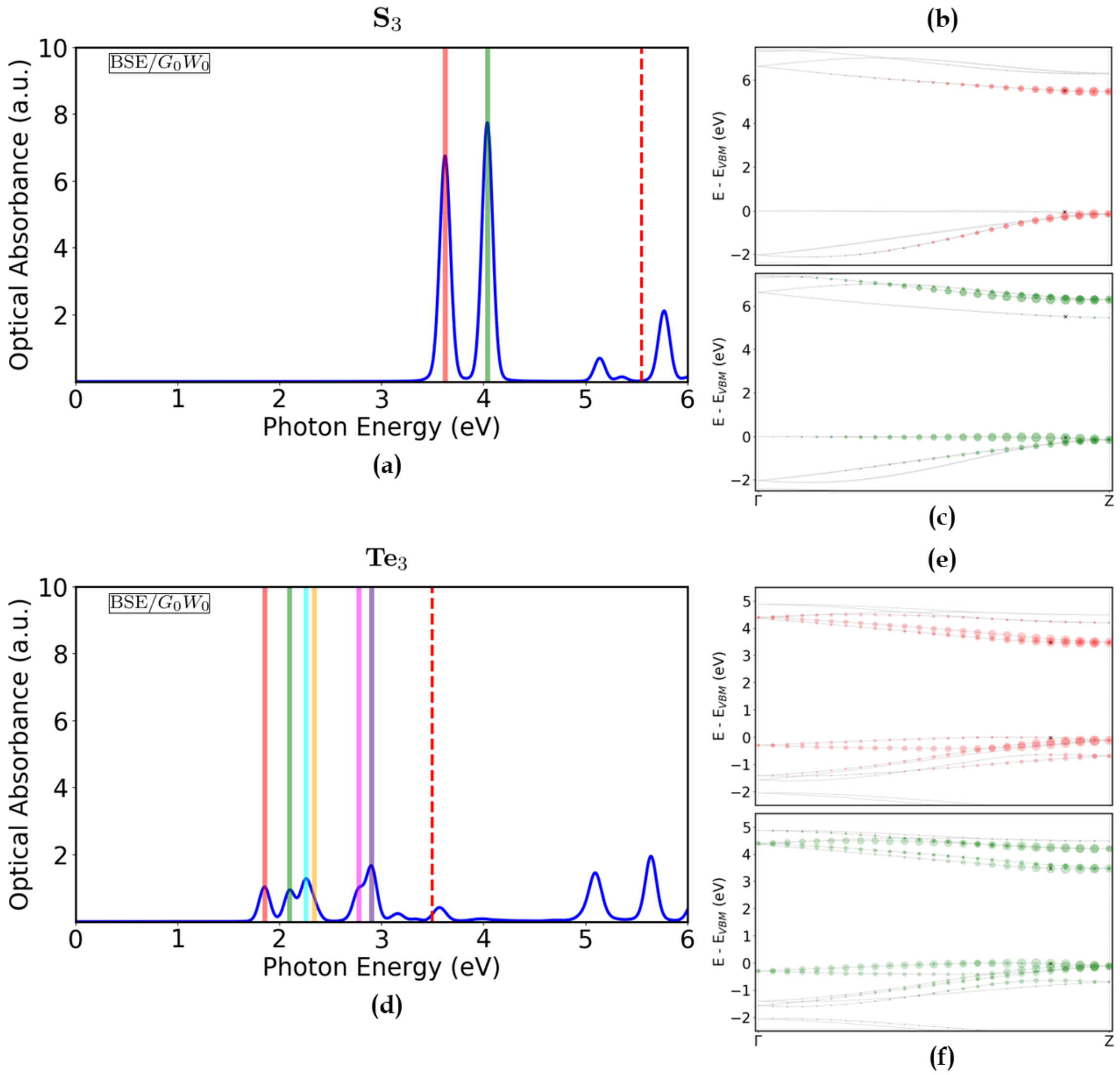


Figure 6: [Left] Absorption spectra (solid blue) of (**a**) $S_3$ and (**d**) $Te_3$, expressed in terms of the optical absorbance $A(\omega)$, calculated at the $G_0W_0$/BSE level. The corresponding $G_0W_0-$corrected direct electronic band gaps (dashed red) are shown as a reference. A broadening of 50 meV was used. [Right] Electronic band structures (solid grey), calculated at the $G_0W_0$ level, of $S_3$ ($\mathbf{b}-\mathbf{c}$) and $Te_3$ ($\mathbf{e}-\mathbf{f}$). The colored dots (red and green) represent the single-particle transitions contributing to the first two bright excitons, and their size is proportional to the intensity of the transition - renormalized to the highest value. The corresponding excitonic peaks are highlighted in the relative absorption spectra (solid red and green), together with other meaningful higher excitations below the electronic gap (see Fig. 8 of Appendix B). Energy zero is set as the top of the valence bands. SOC and semi-core corrections were included.

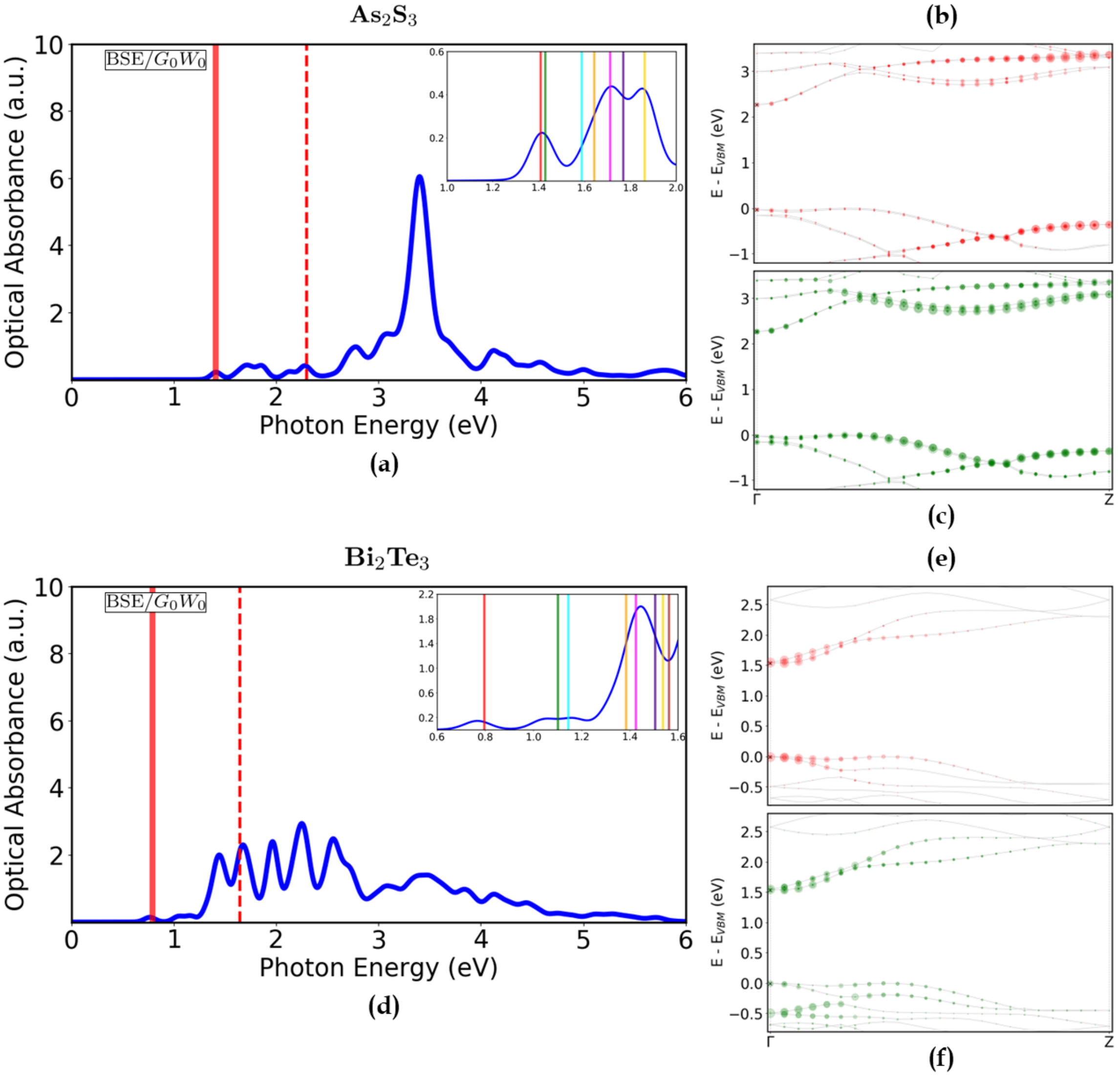


Figure 7: [Left] Absorption spectra (solid blue) of (**a**) $As_2S_3$ and (**d**) $Bi_2Te_3$, expressed in terms of the optical absorbance $A(\omega)$, calculated at the $G_0W_0$/BSE level. The corresponding $G_0W_0-$corrected direct electronic band gaps (dashed red) are shown as a reference. A broadening of 50 meV was used. [Right] Electronic band structures (solid grey), calculated at the $G_0W_0$ level, of $As_2S_3$ ($\mathbf{b}-\mathbf{c}$) and $Bi_2Te_3$ ($\mathbf{e}-\mathbf{f}$). The colored dots (red and green) represent the single-particle transitions contributing to the first two bright excitons, and their size is proportional to the intensity of the transition - renormalized to the highest value. The corresponding excitonic peaks are highlighted in the relative absorption spectra (solid red and green) and in their insets, together with other meaningful higher excitations below the electronic gap (see Figs. 9-10). Energy zero is set as the top of the valence bands. SOC and semi-core corrections were included.

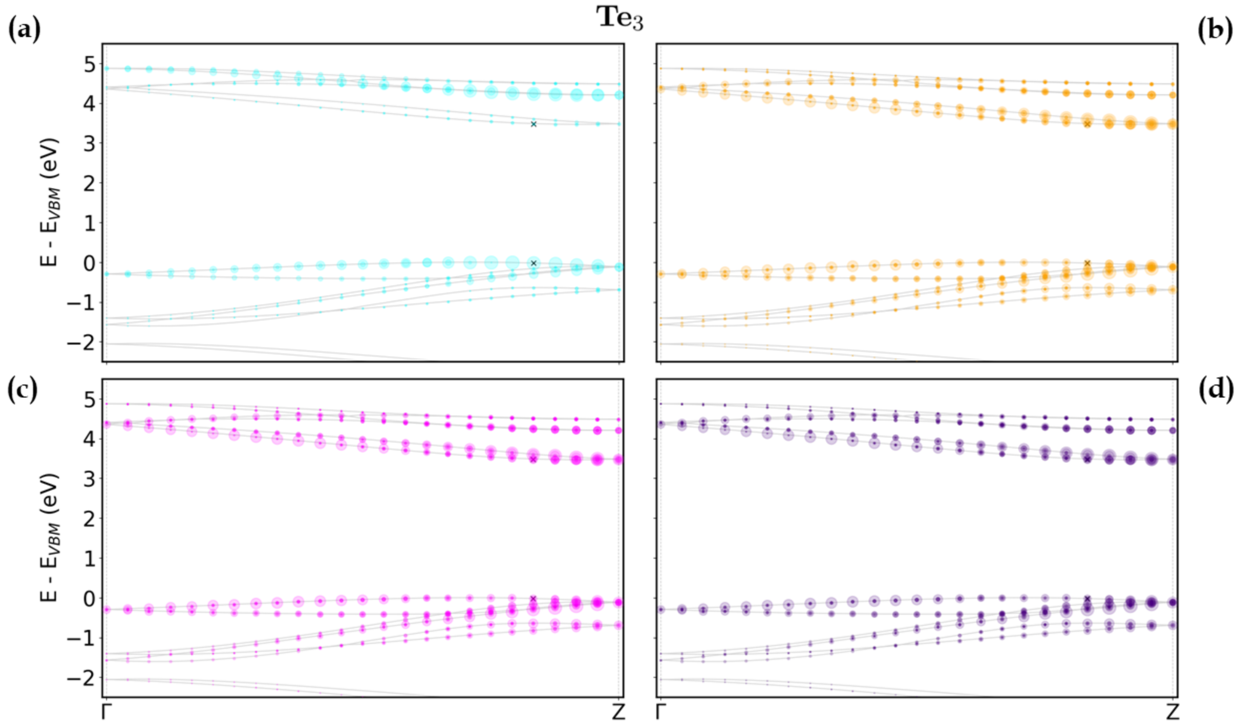


Figure 8: Electronic band structures (solid grey), calculated at the $G_0W_0$ level, of $Te_3$. The colored dots (cyan (**a**), orange (**b**), magenta (**c**) and indigo (**d**)) represent the single-particle transitions contributing to the corresponding bright excitons highlighted in the relative absorption spectrum (Fig. 6 of Appendix B), and their size is proportional to the intensity of the transition - renormalized to the highest value. Energy zero is set as the top of the valence bands. SOC and semi-core corrections were included.

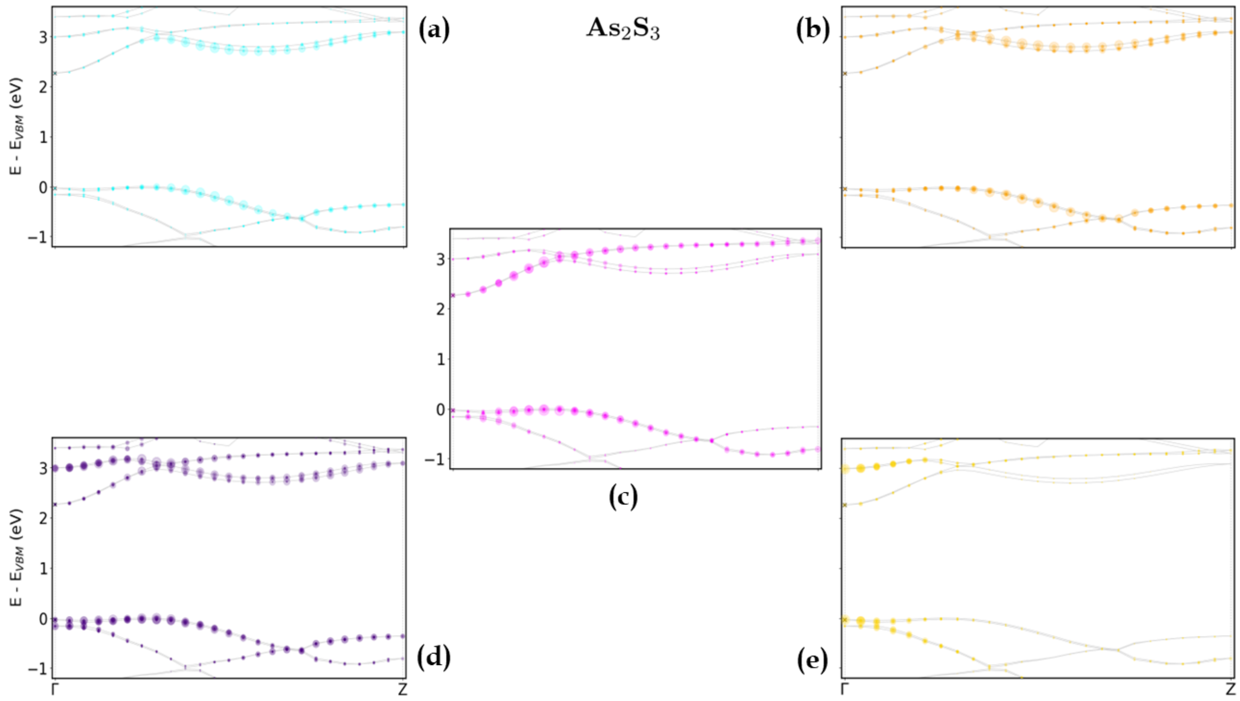


Figure 9: Electronic band structures (solid grey), calculated at the $G_0W_0$ level, of $As_2S_3$. The colored dots (cyan (**a**), orange (**b**), magenta (**c**), indigo (**d**) and gold (**e**)) represent the single-particle transitions contributing to the corresponding bright excitons highlighted in the relative absorption spectrum (Fig. 7), and their size is proportional to the intensity of the transition - renormalized to the highest value. Energy zero is set as the top of the valence bands. SOC and semi-core corrections were included.

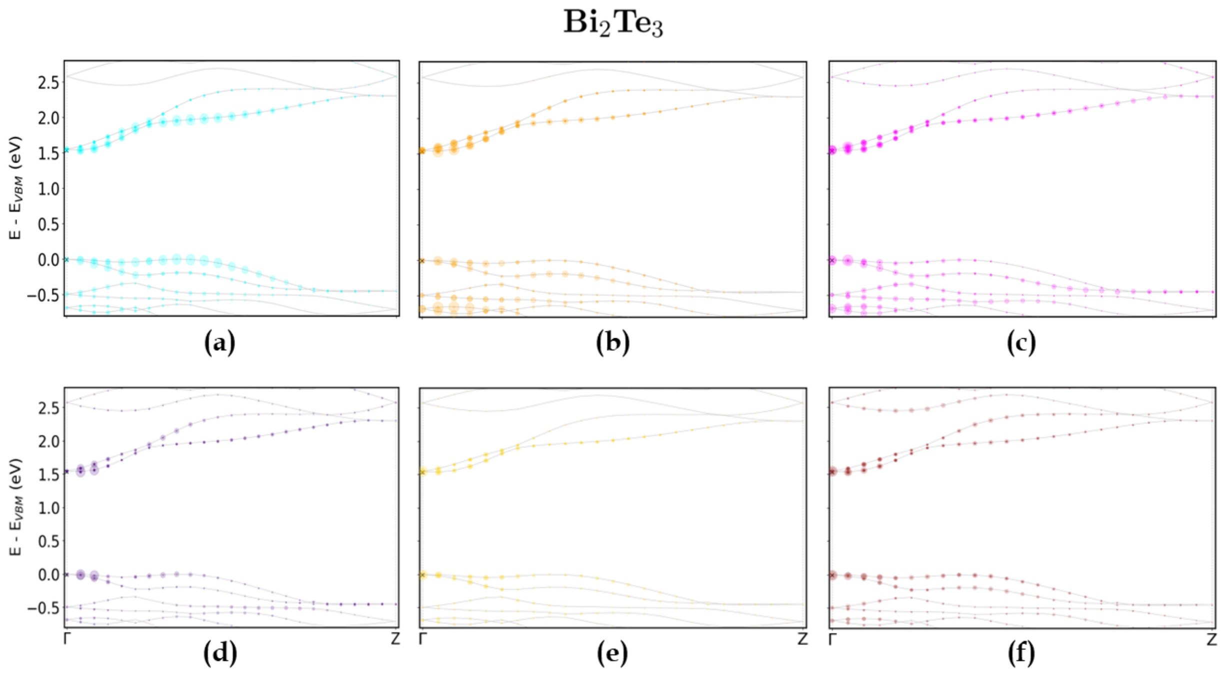


Figure 10: Electronic band structures (solid grey), calculated at the $G_0W_0$ level, of $Bi_2Te_3$. The colored dots (cyan (**a**), orange (**b**), magenta (**c**), indigo (**d**), gold (**e**) and brown (**f**)) represent the single-particle transitions contributing to the corresponding bright excitons highlighted in the relative absorption spectrum (Fig. 7), and their size is proportional to the intensity of the transition - renormalized to the highest value. Energy zero is set as the top of the valence bands. SOC and semi-core corrections were included.

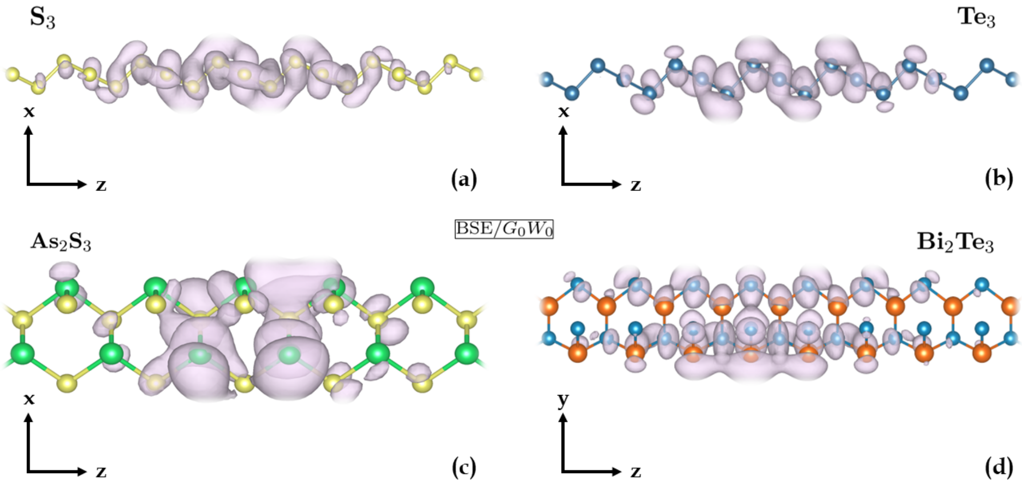


Figure 11: Plots in direct space of the excitonic wavefunctions of the first bright exciton of (**a**) $S_3$, (**b**) $Te_3$, (**c**) $As_2S_3$ and (**d**) $Bi_2Te_3$, calculated at the $G_0W_0$/BSE level. The position of the hole was chosen based on the localization of the valence electrons contributing to the exciton. The number of cell repetitions in the periodic direction (7, 7, 5, 8) was increased until the wavefunction decayed to zero.

## Electronic band structures at the evGW level

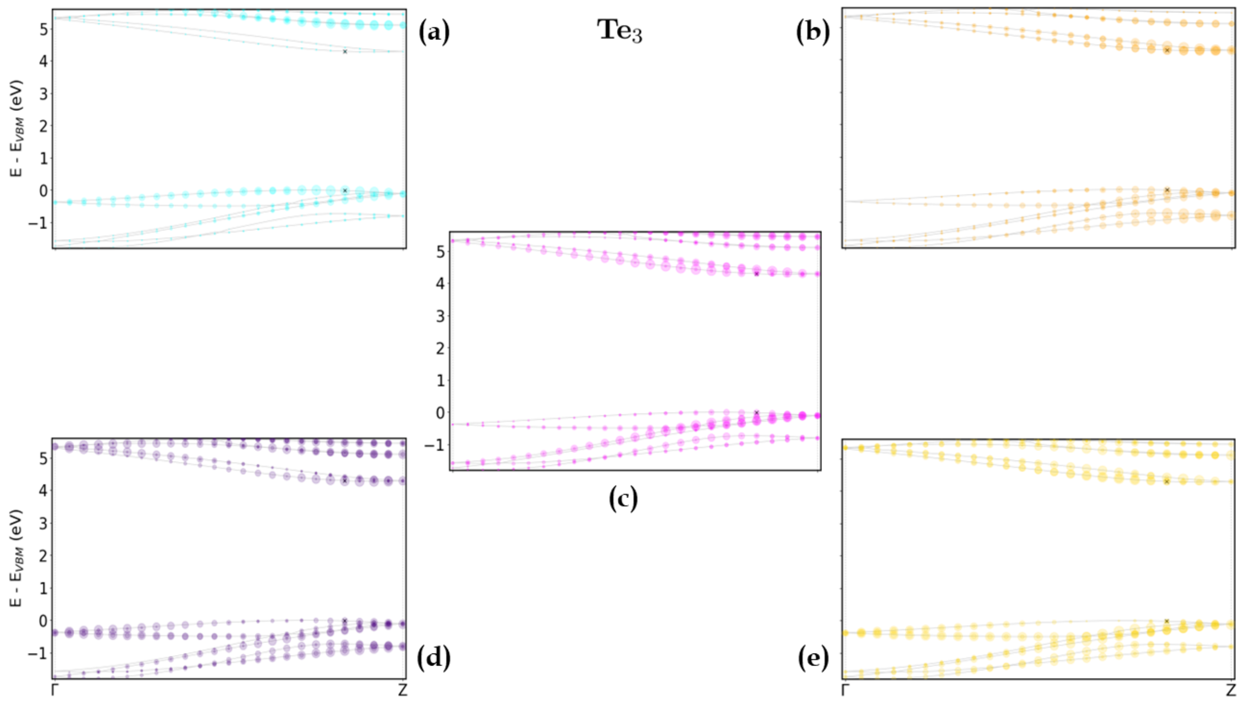


Figure 12: Electronic band structures (solid grey), calculated at the ev*GW* level, of $Te_3$. The colored dots (cyan (**a**), orange (**b**), magenta (**c**), indigo (**d**) and gold (**e**)) represent the single-particle transitions contributing to the corresponding bright excitons highlighted in the relative absorption spectrum (Fig. 6), and their size is proportional to the intensity of the transition - renormalized to the highest value. Energy zero is set as the top of the valence bands. SOC and semi-core corrections were included.

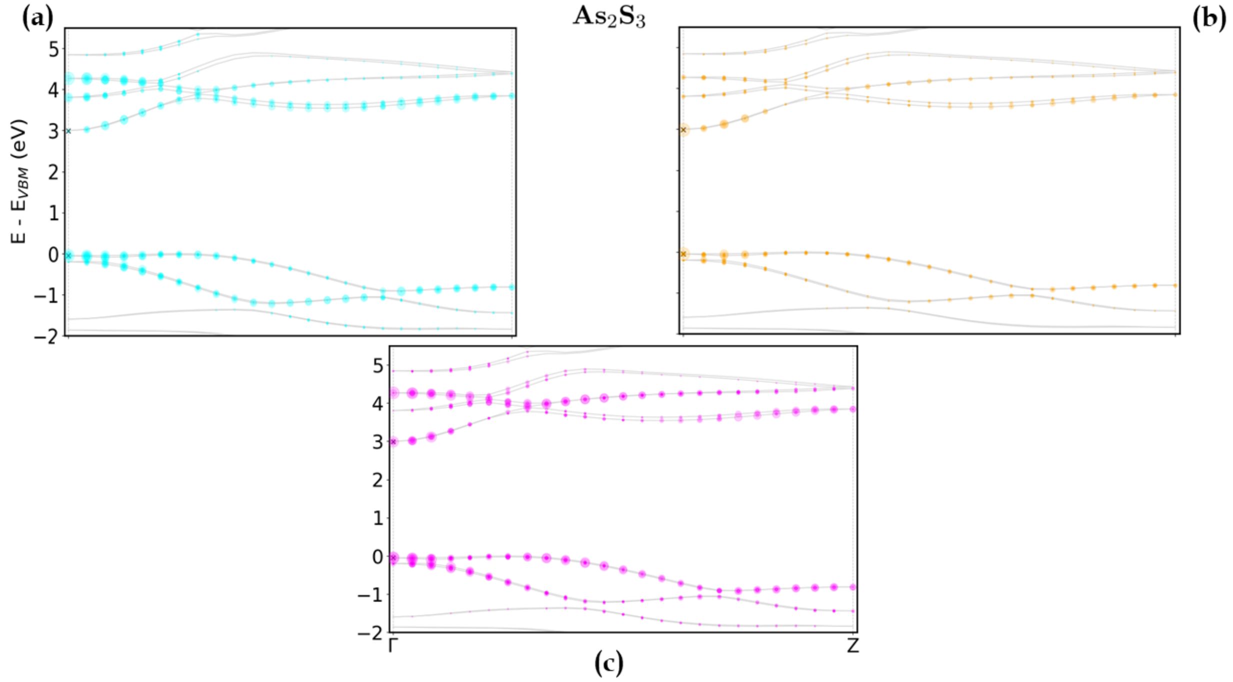


Figure 13: Electronic band structures (solid grey), calculated at the ev$GW$ level, of $As_2S_3$. The colored dots (cyan (**a**), orange (**b**) and magenta (**c**)) represent the single-particle transitions contributing to the corresponding bright excitons highlighted in the relative absorption spectrum (Fig. 7 of the main text), and their size is proportional to the intensity of the transition - renormalized to the highest value. Energy zero is set as the top of the valence bands. SOC and semi-core corrections were included.

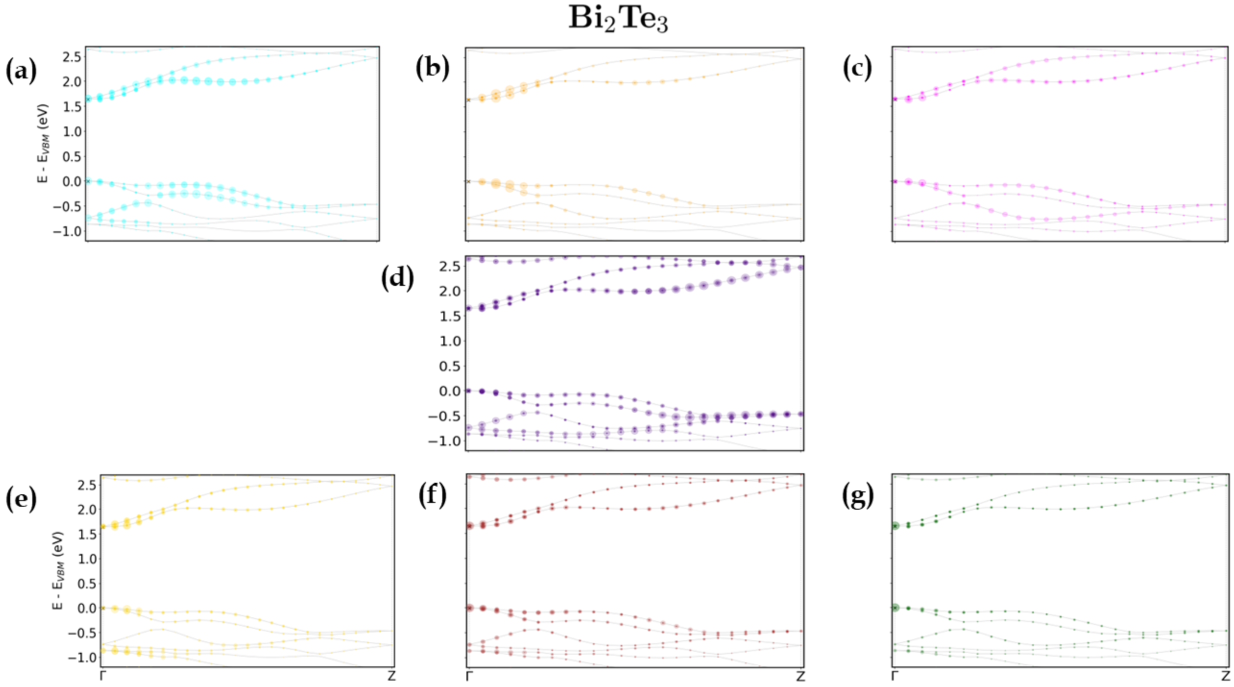


Figure 14: Electronic band structures (solid grey), calculated at the ev$GW$ level, of $Bi_2Te_3$. The colored dots (cyan (**a**), orange (**b**), magenta (**c**), indigo (**d**), gold (**e**), brown (**f**) and dark green (**g**)) represent the single-particle transitions contributing to the corresponding bright excitons highlighted in the relative absorption spectrum (Fig. 7 of the main text), and their size is proportional to the intensity of the transition - renormalized to the highest value. Energy zero is set as the top of the valence bands. SOC and semi-core corrections were included.

# References

(1) Rivano, N.; Marzari, N.; Sohier, T. Density functional perturbation theory for one-dimensional systems: Implementation and relevance for phonons and electron-phonon interactions. *Phys. Rev. B* **2024**, *109*, 245426.

(2) Sohier, T.; Calandra, M.; Mauri, F. Density functional perturbation theory for gated two-dimensional heterostructures: Theoretical developments and application to flexural phonons in graphene. *Phys. Rev. B* **2017**, *96*, 075448.
